# Assessing the Integrated Community-Based Health Systems Strengthening initiative in northern Togo: a pragmatic effectiveness-implementation study protocol

**DOI:** 10.1186/s13012-019-0921-3

**Published:** 2019-10-16

**Authors:** Molly E. Lauria, Kevin P. Fiori, Heidi E. Jones, Sesso Gbeleou, Komlan Kenkou, Sibabe Agoro, Abdourahmane Diparidé Agbèrè, Kelly D. Lue, Lisa R. Hirschhorn

**Affiliations:** 1 Community Health Systems Lab, Integrate Health/Santé Intégrée, Kara, Togo; 2 Department of Pediatrics, Albert Einstein College of Medicine, Bronx, NY USA; 3 Department of Family and Social Medicine, Albert Einstein College of Medicine, Bronx, NY USA; 4 CUNY Graduate School of Public Health & Health Policy, New York, USA; 5 Integrate Health/Santé Intégrée, Kara, Togo; 6 Kara Regional Health Department, Ministry of Health and Public Hygiene, Kara, Togo; 7 Department of Pediatrics, Health Sciences Faculty, University of Lomé, Lomé, Togo; 8 Department of Pediatrics, Regional Hospital, Lomé-Commune, Lomé, Togo; 9 Northwestern University Feinberg School of Medicine, Chicago, USA

**Keywords:** Reproductive, Maternal, Child Health, RE-AIM, Health systems, Community health workers, Supportive supervision, IMCI, iCCM, Togo

## Abstract

**Background:**

Over the past decade, prevalence of maternal and child morbidity and mortality in Togo, particularly in the northern regions, has remained high despite global progress. The causes of under-five child mortality in Togo are diseases with effective and low-cost prevention and/or treatment strategies, including malaria, acute lower respiratory infections, and diarrheal diseases. While Togo has a national strategy for implementing the integrated management of childhood illness (IMCI) guidelines, including a policy on integrated community case management (iCCM), challenges in implementation and low public sector health service utilization persist. There are critical gaps to access and quality of community health systems throughout the country. An integrated facility- and community-based initiative, the Integrated Community-Based Health Systems Strengthening (ICBHSS) initiative, seeks to address these gaps while strengthening the public sector health system in northern Togo. This study aims to evaluate the effect and implementation strategy of the ICBHSS initiative over 48 months in the catchment areas of 21 public sector health facilities.

**Methods:**

The ICBHSS model comprises a bundle of evidence-based interventions targeting children under five, women of reproductive age, and people living with HIV through (1) community engagement and feedback; (2) elimination of point-of-care costs; (3) proactive community-based IMCI using community health workers (CHWs) with additional services including family planning, HIV testing, and referrals; (4) clinical mentoring and enhanced supervision; and (5) improved supply chain management and facility structures. Using a pragmatic type II hybrid effectiveness-implementation study, we will evaluate the ICBHSS initiative with two primary aims: (1) determine effectiveness through changes in under-five mortality rates and (2) assess the implementation strategy through measures of reach, adoption, implementation, and maintenance. We will conduct a mixed-methods assessment using the RE-AIM (reach, effectiveness, adoption, implementation, maintenance) framework. This assessment consists of four components: (1) a stepped-wedge cluster randomized control trial using a community-based household survey, (2) annual health facility assessments, (3) key informant interviews, and (4) costing and return-on-investment assessments for each randomized cluster.

**Discussion:**

Our research is expected to contribute to continuous quality improvement initiatives, optimize implementation factors, provide knowledge regarding health service delivery, and accelerate health systems improvements in Togo and more broadly.

**Trial registration:**

ClinicalTrials.gov, NCT03694366, registered 3 October 2018

**Electronic supplementary material:**

The online version of this article (10.1186/s13012-019-0921-3) contains supplementary material, which is available to authorized users.

Contributions to the literature

- This protocol demonstrates how to utilize implementation science methodologies to conduct a pragmatic trial in a low-income setting in order to improve service delivery and apply findings into practice.
- It provides a model for embedded implementation research in public sector service delivery to facilitate demand-driven research and adoption of scientific findings into policy implementation. Close collaboration and ownership amongst practitioners, policymakers, and researchers are crucial to address research questions for policy change of empirical value to local communities.
- This study will evaluate the effectiveness and implementation strategy of an integrated facility- and community-based initiative within a low-income health system and aims to provide generalizable evidence to policymakers that inform national community health strategy decisions.

## Background

While Togo has observed reductions in child mortality over the last few decades, accelerated progress is needed to achieve Sustainable Development Goal (SDG) goal 3.2, to reduce neonatal mortality to 12 per 1000 live births and under-five mortality to 25 per 1000 live births [1, 2]. Most recent subnational estimates from 2013-2014 show that the infant mortality rate in the northern region of Kara is 62 per 1000 live births and the under-five mortality rate is 130 per 1000 live births, compared to national rates of 49 and 88 per 1000 live births, respectively [3]. The principal causes of under-five deaths in Togo are diseases with effective and low-cost interventions and treatments, including malaria, acute lower respiratory infections, and diarrheal diseases [3]. Despite Togo’s national plan for the integrated management of childhood illness (IMCI), a policy on integrated community case management (iCCM), and the Expanded Program on Immunization (EPI), Togo has not observed comparable reductions in child mortality as compared to neighboring peer nations including Burkina Faso, Ghana, and Guinea [4]. Furthermore, Togo’s Ministry of Health (MoH) reports that national utilization rates of public sector health facilities are less than 30%, even though 62% of the population lives within 5 km of these facilities [5, 6].

### Context and Integrated Community-Based Health Systems Strengthening model

**Fig. 1:**
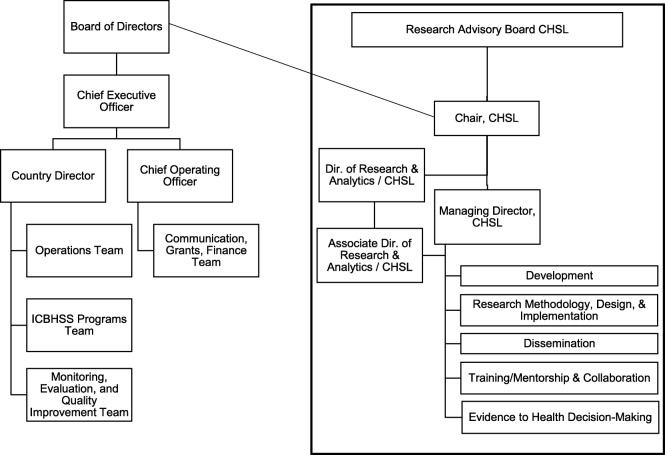
Community Health Systems Lab (CHSL) organization structure within Integrate Health (IH) organizational chart

Integrate Health (IH) is a non-governmental organization (NGO) working in collaboration since 2004 with the Togolese MoH and community-based organizations (CBOs). This public-private partnership focuses on integrating community and public sector health services in the Kara region, initially focusing on HIV [7, 8] and subsequently expanding to primary care [9, 10]. In pursuit of an overarching research objective to improve community health systems through scientific methods, IH formed the Community Health Systems Lab (CHSL), which embeds implementation research and dissemination into IH operations including the current study protocol. See Fig. 1 for CHSL organizational details. IH aims to foster a learning health system approach, through locally produced evidence to both strengthen the effectiveness of our primary partner, the Togolese MoH, and contribute to knowledge generation and uptake by dissemination with local, national, and global colleagues.

In 2014, IH developed a new partnership with the MoH to improve primary healthcare services by addressing the leading causes of premature mortality in the catchment areas of four public sector clinics in the Kozah district of the northern Kara region. These preliminary integrated facility- and community-based health interventions are referred to as the Integrated Community-Based Health Systems Strengthening (ICBHSS) model [11]. This model includes a bundle of evidence-based interventions adapted for implementation in Togo and is consistent with global best practices including the recent World Health Organization (WHO) community health worker (CHW) guideline [12]. The target population includes children under five, pregnant/post-partum women, women of reproductive age for family planning services, and people living with HIV infections. As detailed in Table 1, the ICBHSS model consists of: (1) community engagement and feedback [13–15]; (2) elimination of point-of-care costs for the target population [16–19]; (3) proactive community-based IMCI using salaried CHWs with additional services including family planning and counseling, HIV testing, and referrals [11, 19–27]; (4) clinical mentoring and enhanced supervision at health centers [28–30]; and (5) facility and supply chain management [15, 31–33].

**Table 1:** Overview of Integrated Community-Based Health Systems Strengthening (ICBHSS) model components

	ICBHSS model component	Details
1.	Community engagement meetings and feedback *Evidence:* [13–15]	- Pre-intervention consultation meetings with community leaders. - Community participation in CHW selection. - Biannual community review meetings with local leaders, community members, facility, and IH staff. - IH staff presentation of programmatic results and updates. - Community-provided feedback on ICBHSS implementation challenges, successes, and areas for improvement.
2.	Removal of point-of-care costs in IH intervention public sector health facilities *Evidence:* [16–19]	- Pertains to all children under five, pregnant/post-partum women, women of reproductive age for family planning services, and people living with HIV infections who seek care at study sites. - Includes facility-based consultation fees, medications, supplies, and services provided at IH intervention sites and advanced care referrals at the district or regional hospital. - Selected fees and coverage population chosen in consultation with MoH and based on national and global guidelines.
3.	Proactive community-based IMCI using trained, equipped, supervised, and salaried CHWs with additional services including linkage to family planning and counseling, HIV testing, and referrals *Evidence:* [11, 19–27]	- Candidate selection from community by local leadership, health facility, and IH staff. - Preference for female residents who meet selection criteria (some literacy, pre-test/post-test results, demonstrated related competencies). - Pre-service 23-day training in IMCI, maternal health, and HIV counseling and testing led by MoH and IH staff. - In-service 5-day training in family planning and counseling led by MoH and IH staff. - All training materials developed in consultation with MoH and based on national/global guidelines and evidence-based materials from Association Togolaise pour le Bien-Être Familial, Better Birth Project, Last Mile Health, Muso, and Partners In Health. - Equipped with materials (training guides, backpacks, timers, thermometers, scales, MUACs, rapid tests, medical treatment for basic IMCI cases, notebooks, pens). - CWH consultations, referrals, medicines, and materials are provided free of charge. - Supportive supervision with coaching and mentoring by IH supervisor (nurse/medical assistant). - Regular observation of CHW service delivery through routine programmatic data and community feedback. - CHWs receive a regular equitable salary for full-time work through proactive case seeking and follow-up.
4.	Clinical mentoring and enhanced supervision by a trained peer coach at public sector health facilities *Evidence:* [28–30]	- Onsite pre-service 4-day training in maternal, reproductive, neonatal, and child health and HIV led by IH clinical mentor (nurse/medical assistant) and medical director. - Training materials developed in collaboration with MoH and based on national/global guidelines and evidence-based materials from WHO, American Academy of Pediatrics, Ariadne Labs, Better Birth Project, Last Mile Health, Muso, and Partners In Health. - Weekly facility-based supportive supervision by IH clinical mentors (nurses, midwives, medical assistants) with prior experience in public sector health facilities. - Regular observation of facility staff service delivery through routine programmatic data and community feedback.
5.	Basic infrastructure/equipment improvements and supply chain management training of pharmacy managers *Evidence:* [15, 31–33]	- Formal infrastructure assessment and equipment needs with MoH using WHO’s SARA tool [41]. - Facilitate structural improvements to improve care delivery. - Equip facility with essential medicines and equipment identified by assessment and national health protocols. - Onsite training in supply-chain management practices, including proper storage of medicines, filling of stock cards, and orders based on average monthly input consumption. - Regular supervision and support by IH clinical mentor.

Preliminary results from the ICBHSS implementation pilot in the Kozah district observed a decline in under-five mortality and increased health service utilization for child and maternal health services at all four intervention sites (ClinicalTrials.gov Identifier: NCT03773913). This evaluation was conducted using a repeated, population-representative, cross-sectional household survey. We anticipate results from 48 months of implementation in 2019.

Following this successful preliminary pilot phase and considering the need, IH was requested by the MoH to expand the ICBHSS model to additional sites starting in 2018. In collaboration with the MoH and technical partners, IH is replicating the ICBHSS model in 21 distinct rural health facilities in four additional districts. In collaboration with MoH partners, IH’s ICBHSS program team designed an implementation strategy for this expansion to enhance adoption, implementation, and sustainability of the model [34–36]. This strategy enables a rollout to a new district every 12 months, and is based on pilot experience, local contextual factors as well as budgetary and feasibility considerations. As part of this expansion launched in 2018, IH and MoH partners designed a stepped-wedge randomized trial to enable a rigorous assessment of intervention effectiveness and implementation strategies to inform national policy.

### Rationale for study design

We selected a pragmatic type II hybrid effectiveness-implementation study, as it allows simultaneous mixed-methods assessments of intervention effectiveness and implementation strategies in “real life” health systems settings [35, 37]. Through this convergent design [38], we intend to routinely disseminate [39] our research findings to MoH partners. Effectiveness measures will provide evidence as to whether the ICBHSS intervention impacts child mortality and, via this pragmatic design, will provide more generalizable estimates than traditional study designs [35]. Assessing the implementation strategy will generate knowledge about process outcomes and feasibility including barriers and enabling factors, core components which are generalizable and where local adaptation is needed for replication in other settings. This actionable knowledge is a critical need for implementors in low- and middle-income settings, particularly for complex health systems interventions [12, 40].

Although our preliminary Kozah pilot study provided initial data to suggest ICBHSS’s effectiveness, these results have limited capacity to establish causation due to single-arm design and lack of a valid comparison group. Furthermore, our pilot study was not designed to evaluate implementation strategies required to provide generalizable data that could inform replication and scale activities.

To address these limitations and rigorously assess ICBHSS effectiveness and implementation strategy, we will conduct a summative mixed-methods evaluation using a modified RE-AIM (reach, effectiveness, adoption, implementation, maintenance) framework [42]. This framework is a practical tool that assesses complex interventions in real-world practice including measures of clinical effectiveness and implementation strategy [43]. Effectiveness will be measured using the primary outcome, under-five child mortality, utilizing a stepped-wedge cluster randomized control design. Implementation strategy will be assessed by measures of reach, adoption, implementation, and maintenance of the ICBHSS intervention through data collection with key informants, facility, and the general catchment populations [44]. This adapted evaluation framework will enhance traditional stepped-wedge design by including measures of implementation fidelity [45].

Given the baseline population need, preliminary effectiveness results, and MoH request for scale-up, we deem it unethical to withhold the intervention from any of the comparison sites. Logistical and financial constraints to launch the ICBHSS model already require the sequential implementation needed for a stepped-wedge trial. This protocol is the best fit considering the limitations from the initial pilot using a single-arm, repeated cross-sectional design and the need for generalizable findings to support translating results into practice [45, 46]. Annual data collection, while an increased demand of resources and cost, was deemed both feasible and beneficial, as it will allow regular quantitative and qualitative data analysis and iterative dissemination of results, internally to support programmatic improvements, and with MoH partners for embedded research practices [47]. Our objective in this paper is to describe the research protocol of this pragmatic hybrid effectiveness-implementation study to measure the effectiveness and implementation strategy of the ICBHSS initiative over 48 months in rural northern Togo using an adapted RE-AIM framework.

## Methods/design

### Study aims

The objective of this study is to optimize ICBHSS model implementation using the adapted RE-AIM evaluation framework. To achieve this, we have two primary objectives: (1) to determine the effectiveness of the ICBHSS model and (2) to assess the implementation strategy through measurements of reach, adoption, implementation, and maintenance. Our specific study aims include the following:

- Primary aim 1: effectiveness
  1. Analyze longitudinal changes in maternal and child mortality and morbidity, quality of care parameters, and public sector facility readiness in catchment areas.
- Primary aim 2: implementation strategy
  1. Identify barriers and facilitators contributing to access and quality of ICBHSS services;
  2. Measure changes in coverage, health service utilization rates, and intervention adoption;
  3. Determine ICBHSS implementation costs and return-on-investment estimates.

### Design

This study uses a pragmatic type II hybrid effectiveness-implementation design [35] to evaluate the two primary aims of effectiveness and implementation strategy by the ICBHSS initiative using a modified RE-AIM implementation science framework [42]. See Additional file 1 for the CONSORT checklist. We will include four distinct study components: (1) a stepped-wedge cluster randomized control trial using a community-based household survey, (2) annual health facility assessments at each selected site, (3) qualitative key informant interviews conducted 1-year post-intervention reception, and (4) annual costing and return-on-investment analyses using the Community Health and Costing Tool [48] and the Lives Saved Tool (LiST) [49]. Further details about each study component are described below with Table 2 summarizing a timeline.

**Table 2:**
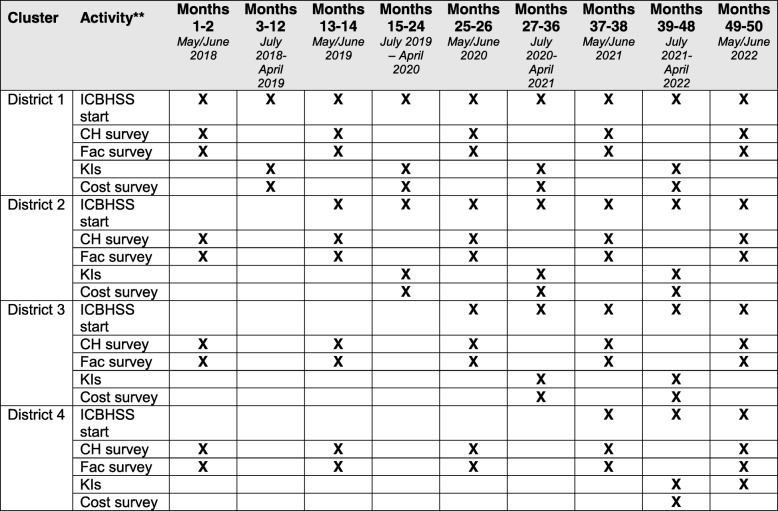
Data collection and Integrated Community-Based Health Systems Strengthening (ICBHSS) initiation timeline based on staggered implementation*

*Follows the CONSORT extension diagram for stepped-wedge cluster randomized trials [52]

**CH community-based household survey, *Fac* health facility assessments, *KIs* key informant interviews, *Cost survey* costing and return-on-investment assessment

#### Stepped-wedge cluster randomized control trial

This pragmatic design leverages the sequential or staggered rollout of the ICBHSS model and will facilitate an assessment of effectiveness as well as implementation strategy through coverage and adoption metrics by comparing geographically organized clusters [50, 51]. It includes four clusters that organize intervention health facilities by district: Bassar, Binah, Dankpen, and Kéran. The ICBHSS cluster initiation order will be randomized annually with four steps, as it was independently determined by the IH programs team that baseline health needs were similar in each cluster. Community-based household surveys adapted from Demographic and Health Survey (DHS) modules previously implemented in Togo [3] and focusing on demographic, maternal, and child health data will be conducted in each cluster at baseline, 12, 24, 36, and 48 months.

#### Health facility assessments

These assessments will employ facility-level surveys based on the World Health Organization (WHO) Service Availability and Readiness Assessment tool (SARA) [41] and will provide effectiveness information about facility-level service quality. Surveys will be completed for each health facility annually at baseline, 12, 24, 36, and 48 months.

#### Key informant interviews

Qualitative interviews will be completed with key informants to assess barriers and facilitators to program implementation fidelity and feasibility while also documenting contextual factors. The first key informant interviews will be conducted 12 months post-intervention start and at subsequent 12-month intervals until study end within each cluster.

#### Costing and return-on-investment assessment

ICBHSS program costs and return on investment will be measured using the Community Health Planning and Costing Tool [48] and the Lives Saved Tool [49] to assess implementation strategy approaches and to inform considerations of maintenance and national planning efforts. The first assessment will be conducted 12 months post-intervention start and at annual subsequent 12-month intervals until study end within each cluster.

### Study setting

The study will be conducted in the catchment areas of 21 public sector health facilities within the Kara region’s rural districts of Bassar, Binah, Dankpen, and Kéran. The total population is approximately 181,111 people. Study sites were selected by MoH partners and IH programmatic staff based on perceived population health needs, ongoing regional public-private program activities, and population size. All selected sites are primary healthcare facilities operated by the MoH [53] that serve rural populations. Estimated catchment population and utilization rates for these sites are listed by district in Table 3. As described in Table 2, the ICBHSS initiative will be sequentially implemented by district each year within these 21 preselected sites.

**Table 3:** List of study sites (*N* = 21) with estimated baseline catchment population (*N* = 181,111) and facility utilization rates

District	Study site	Catchment population*	Facility utilization rate**
Bassar	Bangéli	16,169	42%
Bassar	Kabou-Sara	10,054	56%
Bassar	Koundoum	7,428	37%
Bassar	Manga	5,006	28%
Bassar	Sanda-Afohou	5,514	43%
Binah	Asseré	4,446	19%
Binah	Boufalé	4,212	48%
Binah	Kouyorira	4,364	26%
Binah	N’Djei	3,258	53%
Binah	Pessaré	8,002	26%
Binah	Sirka	5,980	51%
Binah	Solla	5,960	58%
Dankpen	Koutière	13,097	12%
Dankpen	Kpétab	8,208	22%
Dankpen	Naware	19,531	10%
Dankpen	Solidarité	8,157	24%
Kéran	Kokou-Temberma	8,722	Unknown
Kéran	Nadoba	16,593	Unknown
Kéran	Natiponi	7,636	Unknown
Kéran	Pangouda	10,735	Unknown
Kéran	Warengo	8,039	Unknown

*Data derived from 2016 Ministry of Health population estimates and 2018 Integrate Health baseline population-based sampling

**Data source is 2016 Ministry of Health district-level annual reports

### Inclusion criteria

Eligibility for inclusion is described below by study component for the community-based household surveys, facility surveys, and qualitative interviews.

#### Community-based household surveys

Females 15–49 years of age who reside in a selected household within the study catchment area are included. Participants 15–17 years of age will only be included if they have been/are currently pregnant and have a waiver of parental permission. Households will be randomly selected each year using a systematic population-weighted sampling methodology. If there is more than one eligible respondent in the household, one will be randomly selected based on a Kish selection grid [54]. All participants will have the opportunity to decline participation during the informed consent process.

#### Health facility surveys

All 21 selected health facilities will be surveyed. See Table 3 for facility details.

#### Key informant interviews

This component will include individuals 18 years of age or older who are either implementing partners or intervention health facility staff. Implementing partners are defined as IH programmatic staff members employed in the catchment area of interest. Health facility staff are defined as clinical or administrative MoH employees working at one of the 21 health centers. Approximately 42 key informants from the 21 health facilities (21 implementing partners and 21 clinical health facility staff) will be included.

### Sample size determination

This study is powered to detect a change by cluster in under-five mortality per 1000 live births. A sample size of 7600 participants will provide 80% power to detect an estimated 30% reduction or greater of under-five mortality from the estimated baseline of 70 per 1000 live births, with an alpha of 0.05, intracluster correlation of 0.005, 20% non-response rate, and estimated 0.5 children under five per participant [9, 55]. Effect size is a conservative estimate based on past pilot experience (ClinicalTrials.gov Identifier: NCT03773913).

#### Randomization

The cluster order for implementation at each step will be determined randomly by an external technical advisor using a random number generator. Randomization will occur each year 8 months prior to the rollout of the intervention in the next cluster. This will enable blinding to the random order of clusters for IH and MoH staff 7involved in implementation while also allowing for an annual 8-month planning stage prior to start of the intervention. Each cluster represents a district, with a total of 21 preselected health facilities across each of the four districts.

### Data collection and analysis

All metrics will be organized using a modified RE-AIM (reach, effectiveness, adoption, implementation, maintenance) framework [56]. Table 4 summarizes data collection and analysis plans organized by primary aim and adapted RE-AIM domains.

**Table 4:** Summary of key study measures organized by aim and domain using modified RE-AIM evaluation framework

Study aim	RE-AIM framework domain	Outcome(s)/indicator(s)	Data source	Indicator definition/clarification
Study aim I: implementation strategy	Reach	*Health service coverage*		
		CHW home visits	Community-based household survey	Proportion of participants in the last year reporting a home visit by an IH CHW.
		CHW home treatment	Community-based household survey	Proportion of participants in the last year reporting treatment at home by an IH CHW
		Health facility treatment	Community-based household survey	Proportion of participants in the last year reporting care at a health facility.
		*Early service access for child health*		
		Malaria coverage	Community-based household survey	Proportion of children under five reported febrile receiving guideline-based treatment within 24 h of symptom onset.
		Pneumonia coverage	Community-based household survey	Proportion of children under five with a cough and proportion of those receiving guideline-based treatment within 24 h of symptom onset.
		Gastrointestinal illness coverage	Community-based household survey	Proportion of children under five with diarrhea receiving guideline-based treatment within 24 h of symptom onset.
		Malnutrition coverage	Community-based household survey	Proportion of children under five with malnutrition receiving effective treatment.
		Coverage estimate of prenatal care	Community-based household survey	Proportion of pregnant women in the last two years who completed four ANC visits.
		Percentage of births at healthcare facility	Community-based household survey	Proportion of pregnant women in the last two years who delivered in a health facility
		Percentage of births at home	*Community-based household survey*	Proportion of pregnant women who delivered at home in last 2 years.
		Coverage estimate of post-natal care	Community-based household survey	Proportion of post-partum women who received post-natal care in last 2 years.
Study aim 2: effectiveness	Effectiveness	*Primary outcome*		
		Children under five mortality rate	Community-based household survey	Using a standard birth/death history table, calculate under-five mortality rates, and compare risk of death before age five across surveys with the Cox proportional hazards regression using survey year as the explanatory variable. Children still alive and under age five at the time of survey will be right censored.
		*Secondary outcomes*		
		Neonatal mortality rate	Community-based household survey	The neonatal mortality rates from all births reported by respondents in the 5 years prior to the survey using the same methods described for under-five mortality, adjusted for 28 days.
		Children under one mortality rate	Community-based household survey	The under-one mortality rates from all births reported by respondents in the 5 years prior to the survey using the same methods described for under-five, adjusted for 1 year.
		Children under two mortality rate	Community-based household survey	The under-two mortality rates from all births reported by respondents in the 5 years prior to the survey using the same methods described for under-five, adjusted for 2 years.
		Maternal mortality rate	Community-based household survey	Exploratory maternal mortality analysis based on the sisterhood reports [57, 58].
		Quality of care parameters		
		Timeliness/promptitude of child care for malaria	Routine programmatic data	Proportion of children under five reported febrile and the proportion who received effective antimalarial treatment within 24 h of symptom onset.
		Timeliness/promptitude of child care for pneumonia	Routine programmatic data	Proportion of children under five reported with cough and the proportion of those children who received an effective pneumonia treatment within 24 h of symptom onset.
		Timeliness/promptitude of child care for diarrhea	Routine programmatic data	Proportion of children under five reported with diarrhea and the proportion of those children who received an effective treatment for diarrheal disease within 24 h of symptom onset.
		CHW technical competence	Routine programmatic data	Proportion of IH CHWs who adhere to evidence-based protocols for iCCM and maternal health.
		Facility clinical staff technical competence	Routine programmatic data	Proportion of facility clinical staff who adhere to evidence-based protocols for iCCM and maternal health.
		Equitable	Community-based household survey	Access differences in child mortality between maternal wealth quintiles, distance to facility, and education level.
		Healthcare readiness score	Health facility assessments	Examine facility changes in procurement, physical infrastructure, and management through annual readiness score [41].
Study aim I : implementation strategy	Adoption	*Community-level engagement*		
		Community engagement sessions	Routine programmatic data	Number of community forums and community members in attendance.
		*Participant-level behavior change*		
		Child care cascades for fever, pneumonia, and diarrhea	Community-based household survey	Changes in childcare-seeking behavior over time for fever, pneumonia, and diarrhea in patients presenting to health clinic, CHW, or non-clinical site. Test if these proportions increased using the same approach to mixed-effects generalized linear models as described in the primary effectiveness outcome measure.
		Women of reproductive age cascade for antenatal care, facility-based delivery, and post-natal care	Community-based household survey	Changes in pregnant women care-seeking behavior over time, services delivered by IH CHWs, facility-based delivery, as well as antenatal care and post-natal care attendance. Test if these proportions increased using the same approach to mixed effects generalized linear models as described in the primary effectiveness outcome measure.
Study aim I : implementation strategy	Implementation	*Qualitative interviews*		
		Fidelity	Key informant interviews	Degree that intervention(s) were implemented as planned in original protocol.
		Feasibility	Key informant interviews	Extent that an intervention can be carried out in a particular setting.
		Outer context [59]	Key informant interviews	Macro-level external factors including social, funding, and leadership.
		Inner context [59]	Key informant interviews	Micro-level internal factors including IH/MoH partnership, distinct issues about IH and MoH roles, feedback, facility, community, household, and individual level.
Study aim I : implementation strategy	Maintenance	*Costing and return-on-investment assessment*		
		Annual price per capita	Costing surveys	Price per capita compared to current MoH funding using the Community Health Planning and Costing Tool [48] at the cluster level.
		Return on investment	Costing surveys	Return on investment using the Community Health Planning and Costing Tool [48] and the Lives Saved Tool [49] with primary outcome at the cluster level.

#### Primary aim 1: effectiveness

##### Effectiveness

We define *effectiveness* metrics as those that assess the impact of the ICBHSS initiative using annual community household surveys, routine programmatic data, and the health facility assessments. The community-level primary outcome by district uses under-five mortality rates as well as the secondary outcomes of neonatal, under-one, under-two, and maternal mortality rates. We will additionally evaluate quality of care parameters focusing on timeliness of child healthcare through promptitude of treatment reception following illness diagnosis and health facility readiness scores. Lastly, we will assess equity through secondary analyses of under-five mortality by household wealth quintiles, maternal education levels, and distance from the nearest health facility.

*Primary outcome: under-five mortality rates*. We will calculate under-five mortality rates from all births reported by respondents using a standard birth/death history table. We will calculate under-five mortality rates and compare the risk of death before age five across surveys with the Cox proportional hazards regression using intervention exposure as the explanatory variable. Children still alive and under age five at the time of survey will be right censored.

*Secondary outcomes: neonatal, under-one, and under-two mortality rates*. We will calculate the neonatal, under-one, and under-two mortality rates from all births reported by respondents using the same methods described above adjusted for 28 days, 1 year, and 2 years.

*Secondary outcome: maternal mortality*. We will calculate an exploratory maternal mortality analysis based on sisterhood reports [57, 58].

#### Primary aim 2: implementation strategy

##### Reach

We define *reach* metrics as the proportion of target population that gained access to the ICBHSS initiative services using the annual community household surveys. We will assess the implementation strategy through individual-level ICBHSS participation with community-level (vis-à-vis CHWs) and facility-level health service utilization using health service coverage estimates and early service access for child health. We will use a mixed-effects generalized linear model to compare pre-intervention to post-intervention proportions for each *reach* metric while adjusting for clustering at the facility and district level and time and allowing for district-level estimates to be random effects. Our primary analysis will not include adjustment for individual-level characteristics, as each district will serve as its own control.

##### Adoption

We define *adoption* metrics for this study as the proportion of the community and providers changing health-seeking or providing behavior. Our evaluation of *adoption* will be completed using routine programmatic data and annual community household surveys. Through this domain, we will assess implementation strategy measures using community-level engagement and behavior change by the individual-level uptake of the ICBHSS intervention. We will test whether these proportions increased, applying the same approach described in the *reach* evaluation measures through mixed-effects generalized linear models.

##### Implementation

Metrics of *implementation* are expressed as fidelity and feasibility as well as documenting contextual factors [59]. Evaluation of *implementation* will be completed using key informant qualitative assessments (in-depth interviews with implementing partners and health facility staff) that will be conducted 1-year post-intervention at the cluster (district) level. It will complement quantitative data collected to evaluate implementation strategy and will assess emerging themes.

##### Maintenance

Our study defines *maintenance* metrics as costs required to deliver and sustain the ICBHSS model. Evaluation of *maintenance* will be completed using the costing and return-on-investment analysis, which will be conducted each year of implementation at the cluster (district) level. This analysis will assess program implementation costs based on strategy design retrospectively using the Community Health Planning and Costing Tool [48] and the Lives Saved Tool [49]. These results will be used to inform planning and policy decisions and processes. Lastly, to further triangulate the validity of our findings, we will compare our baseline and 36-month estimates for the domains of *reach*, *effectiveness*, and *adoption* to the most recent Togo DHS or Multiple Indicator Cluster Survey (MICS) data. We will furthermore compare the domains of *reach* and *adoption* to ICBHSS through programmatic data collected at the community and facility level.

### Dissemination plan

We will routinely disseminate study data with key stakeholders in Togo at the national, subnational, and community level, as well as the global community of public health practitioners, researchers, and policymakers. IH staff will conduct biannual forums with local leaders, public sector health facility staff, and community members to discuss ICBHSS implementation and share findings. Based on these forums, IH and MoH partners will collaboratively decide to adapt the implementation strategy and, if deemed necessary, the intervention. IH staff will additionally participate in MoH monthly district-level meetings for district health personnel to stay current in MoH plans and to share findings from ICBHSS initiatives. Results will be published in conference abstracts and peer-reviewed journals with preference for publicly available publications in collaboration with partners at the Togolese MoH.

## Discussion

We have described our rationale, study design, and implementation strategy details regarding this pragmatic type II hybrid design to serve as a model for those interested in pragmatic implementation studies that allow for continuous intervention improvement.

This study design includes several limitations, including limitations with any real-world pragmatic stepped-wedge trial. There are concerns related to confounding, bias, and temporal trends that may limit the validity of our findings. We used a modest effect size, a cluster randomization, and an analysis plan to mitigate these limitations. There are contextual factors that may be challenging to delineate that influence our primary outcomes. We attempted to minimize this at baseline through site selection.

In spite of these limitations, this study will enable a rigorous evaluation in a real-world setting that measures effectiveness and implementation strategy, while also contributing to knowledge generation to inform and complement national health strategies. A one-size-fits-all approach for evaluation does not work for the implementation of multiple interventions and corresponding strategies, particularly in real-world settings. We intend to provide an effective intervention accompanied by an implementation roadmap that includes enabling and non-enabling factors. Through this directed scientific inquiry and mixed-methods evaluation, we aim to contribute to knowledge and foster partnerships that improve quality and access to community health systems in Togo and beyond.

## Additional file

Additional file 1: CONSORT checklist of information to include when reporting a stepped-wedge cluster randomized trial (SW-CRT). (DOCX 18 kb)

Additional file 2: French translation of manuscript text with included figures and tables. (DOCX 218 kb)

## Data Availability

The associated study protocol and data collection tools will be made available upon request from the corresponding author. Quantitative datasets are available from the corresponding author upon reasonable request after the completion of primary analyses and results dissemination. Qualitative study datasets will not be available, as they may include identifiable information that could comprise participant identity.

## Abbreviations

CBO: Community-based organization
CHSL: Community Health Systems Lab
CHW: Community health worker
DHS: Demographic and Health Survey
EPI: Expanded Program on Immunization
ICBHSS: Integrated Community-Based Health Systems Strengthening model
iCCM: Integrated Community Case Management
IH: Integrate Health
IMCI: Integrated Management of Childhood Illness
LiST: Lives Saved Tool
MICS: Multiple Indicator Cluster Surveys
MoH: Ministry of Health and Public Hygiene
NGO: Non-governmental organization
RE-AIM: Reach, Effectiveness, Adoption, Implementation, Maintenance Framework
SARA: Service Availability and Readiness Assessment
SDG: Sustainable Development Goal
SW-CRT: Stepped-wedge cluster randomized trial
WHO: World Health Organization
