# Supplementary material for: Assessing the Integrated Community-Based Health Systems Strengthening initiative in northern Togo: a pragmatic effectiveness-implementation study protocol
Source: Implement Sci. 2019 Oct 16;14:92. doi: 10.1186/s13012-019-0921-3 (PMC6796416; doi:10.1186/s13012-019-0921-3)
Supplement: Supplementary file 1 — CONSORT checklist of information to include when reporting a stepped-wedge cluster randomized trial (SW-CRT). (DOCX 18 kb) [file 13012_2019_921_MOESM1_ESM.docx]

Additional file 1: CONSORT checklist of information to include when reporting a stepped-wedge cluster randomized trial (SW-CRT)

| **Topic** | **Item no** | **Checklist item** | **Page no** |
| --- | --- | --- | --- |
| **Title and abstract** | | | |
|  | 1a | Identification as a stepped-wedge cluster randomized trial in the title. | Not applicable (Abstract) |
|  | 1b | Structured summary of trial design, methods, results, and conclusions (see separate SW-CRT checklist for abstracts). | Page 1 (Abstract) |
| **Introduction** | | | |
| Background and objectives | 2a | Scientific background. Rationale for using a cluster design and rationale for using a stepped-wedge design. | Pages 4-5 (Introduction Study Design & Rationale*)* |
|  | 2b | Specific objectives or hypotheses. | Page 5 (Methods/Design Study Aims) |
| **Methods** | | | |
| Trial design | 3a | Description and diagram of trial design including definition of cluster, number of sequences, number of clusters randomized to each sequence, number of periods, duration of time between each step, and whether the participants assessed in different periods are the same people, different people, or a mixture. | Pages 6-7 (Study Design/Setting) & Table 2 |
|  | 3b | Important changes to methods after trial commencement (such as eligibility criteria), with reasons. | Not yet applicable |
| Participants | 4a | Eligibility criteria for clusters and participants. | Page 6 (Study Setting/Inclusion Criteria) |
|  | 4b | Settings and locations where the data were collected. | Page 6 (Study Setting); Table 3 |
| Interventions | 5 | The intervention and control conditions with sufficient details to allow replication, including whether the intervention was maintained or repeated, and whether it was delivered at the cluster level, the individual participant level, or both. | Table 1 |
| Outcomes | 6a | Completely defined prespecified primary and secondary outcome measures, including how and when they were assessed. | Page 7-8 (Data collection & analysis); Table 3 |
|  | 6b | Any changes to trial outcomes after the trial commenced, with reasons. | Not yet applicable |
| Sample size | 7a | How sample size was determined. Method of calculation and relevant parameters with sufficient detail so the calculation can be replicated. Assumptions made about correlations between outcomes of participants from the same cluster. (see separate checklist for SW-CRT sample size items). | Page 7  (Sample Size Determination) |
|  | 7b | When applicable, explanation of any interim analyses and stopping guidelines. | Page 7-8 (Data collection & analysis); Table 3 |
| **Randomization** | | | |
| Sequence generation | 8a | Method used to generate the random allocation to the sequences of treatments. | Page 7 (Randomization) |
|  | 8b | Type of randomization; details of any constrained randomization or stratification, if used. | Not applicable |
| Allocation concealment mechanism | 9 | Specification that allocation was based on clusters; description of any methods used to conceal the allocation from the clusters until after recruitment. | Page 7 (Randomization) |
| Implementation | 10a | Who generated the randomization schedule, who enrolled clusters, and who assigned clusters to sequences. | Page 7 (Randomization) |
|  | 10b | Mechanism by which individual participants were included in clusters for the purposes of the trial (such as complete enumeration, random sampling; continuous recruitment or ascertainment; or recruitment at a fixed point in time), including who recruited or identified participants. | Page 7 (Randomization) |
|  | 10c | Whether, from whom, and when consent was sought and for what; whether this differed between treatment conditions. | Page 6-7 (Inclusion Criteria) |
| Blinding | 11a | If done, who was blinded after assignment to sequences (e.g., cluster level participants, individual level participants, those assessing outcomes) and how. | Not applicable |
|  | 11b | If relevant, description of the similarity of treatments. | Not applicable |
| Statistical methods | 12a | Statistical methods used to compare treatment conditions for primary and secondary outcomes including how time effects, clustering and repeated measures were taken into account. | Page 7-8  (Data Collection & Analysis); Table 4 |
|  | 12b | Methods for additional analyses, such as subgroup analyses, sensitivity analyses, and adjusted analyses. | Page 7-8  (Data Collection & Analysis); Table 4 |
| **Results** | | | |
| Participant flow (a diagram is strongly recommended) | 13a | For each treatment condition or allocated sequence, the numbers of clusters and participants who were assessed for eligibility, were randomly assigned, received intended treatments, and were analyzed for the primary outcome (see separate SW-CRT flow chart). | Table 2 |
|  | 13b | For each treatment condition or allocated sequence, losses and exclusions for both clusters and participants with reasons. | Not yet applicable |
| **Recruitment** | 14a | Dates defining the steps, initiation of intervention, and deviations from planned dates. Dates defining recruitment and follow-up for participants. | Table 2 |
|  | 14b | Why the trial ended or was stopped. | Not yet applicable |
| **Baseline data** | 15 | Baseline characteristics for the individual and cluster levels as applicable for each treatment condition or allocated sequence. | Not yet applicable |
| **Numbers analyzed** | 16 | The number of observations and clusters included in each analysis for each treatment condition and whether the analysis was according to the allocated schedule. | Not yet applicable |
| Outcomes and estimation | 17a | For each primary and secondary outcome, results for each treatment condition, and the estimated effect size and its precision (such as 95% confidence interval); any correlations (or covariances) and time effects estimated in the analysis. | Not yet applicable |
|  | 17b | For binary outcomes, presentation of both absolute and relative effect sizes is recommended. | Not yet applicable |
| Ancillary analyses | 18 | Results of any other analyses performed, including subgroup analyses and adjusted analyses, distinguishing prespecified from exploratory. | Not yet applicable |
| Harms | 19 | Important harms or unintended effects in each treatment condition (for specific guidance see CONSORT for harms). | Not yet applicable |
| **Discussion** | | | |
| Limitations | 20 | Trial limitations, addressing sources of potential bias, imprecision, and, if relevant, multiplicity of analyses | Page 9 (Discussion) |
| Generalizability | 21 |  |  |
| Interpretation | 22 | Interpretation consistent with results, balancing benefits and harms, and considering other relevant evidence | Not yet applicable |
| **Other Information** | | | |
| Registration | 23 | Registration number and name of trial registry | Page 1 (abstract) |
| Protocol | 24 | Where the full trial protocol can be accessed, if available. | Not applicable |
| Funding | 25 | Sources of funding and other support (such as supply of drugs), and the role of funders. | Page 9 |
| Research ethics review | 26 | Whether the study was approved by a research ethics committee, with identification of the review committee(s). Justification for any waiver or modification of informed consent requirements. | Page 10 |
