# Supplementary material for: Assessing the Integrated Community-Based Health Systems Strengthening initiative in northern Togo: a pragmatic effectiveness-implementation study protocol
Source: Implement Sci. 2019 Oct 16;14:92. doi: 10.1186/s13012-019-0921-3 (PMC6796416; doi:10.1186/s13012-019-0921-3)
Supplement: Supplementary file 2 — French translation of manuscript text with included figures and tables. (DOCX 218 kb) [file 13012_2019_921_MOESM2_ESM.docx]

**Titre : évaluer l’initiative de renforcement des systèmes de santé et à base communautaire dans le nord du Togo : un protocole d’étude pragmatique d’efficacité et de mise en œuvre**

Auteurs :

Molly E. Lauria Titulaire d’un master en santé publique*

Community Health Systems Lab, Integrate Health/Santé Intégrée, Kara, Togo et New York, NY, États-Unis

Kevin P. Fiori Docteur en médecine, titulaire d’un master en santé publique et d’une maîtrise en sciences

Service de pédiatrie, Albert Einstein College of Medicine, Bronx, NY, États-Unis ; service de médecine familiale et sociale, Albert Einstein College of Medicine, Bronx, NY, États-Unis ;

Community Health Systems Lab, Integrate Health/Santé Intégrée, Kara, Togo et New York, NY, États-Unis

Heidi E. Jones Titulaire d’un master en santé publique et d’un doctorat

CUNY Graduate School of Public Health & Health Policy, New York, États-Unis

Sesso Gbeleou Titulaire d’une maîtrise en sciences

Integrate Health/Santé Intégrée, Kara, Togo et New York, NY, États-Unis

Komlan Kenkou Docteur en médecine, titulaire d’une maîtrise en sciences dans le domaine de la santé publique

Integrate Health/Santé Intégrée, Kara, Togo et New York, NY, États-Unis

Sibabe Agoro Docteur en médecine

Service sanitaire régional de Kara, ministère de la Santé et de l’Hygiène publique, Kara, Togo

Abdourahmane Diparidé Agbèrè Docteur en médecine

Service de pédiatrie, faculté des sciences de la santé, université de Lomé, Lomé, Togo :

service de pédiatrie, hôpital régional, Lomé-Commune, Lomé, Togo

Kelly D. Lue Titulaire d’un master en santé publique

Community Health Systems Lab, Integrate Health/Santé Intégrée, Kara, Togo et New York, NY, États-Unis

Lisa R. Hirschhorn Docteur en médecine et titulaire d’un master en santé publique

Feinberg School of Medicine de la Northwestern University, Chicago, États-Unis; Community Health Systems Lab, Integrate Health/Santé Intégrée, Kara, Togo et New York, NY, États-Unis

* Coordonnées de l’auteur correspondant [mlauria@integratehealth.org](mailto:mlauria@integratehealth.org)

**Résumé**

**Contexte :** au cours de la dernière décennie, la prévalence de la morbidité et de la mortalité maternelles et infantiles au Togo, en particulier dans les régions du nord, est restée élevée malgré les progrès mondiaux. La mortalité chez les enfants âgés de moins de cinq ans au Togo est due à des maladies pour lesquelles il existe une prévention et/ou des stratégies de traitement efficaces et de faible coût, telles que le paludisme, les infections des voies respiratoires inférieures aiguës et les maladies diarrhéiques. Alors que le Togo dispose d’une stratégie nationale pour la mise en œuvre des directives de la Prise en Charge Intégrée des maladies de l’Enfant (PCIME), incluant une politique sur la prise en charge intégrée des cas au niveau communautaire (PCIME-C), des difficultés liées à la mise en œuvre et à la faible utilisation des services de santé du secteur public persistent. Il existe des lacunes importantes en ce qui concerne la qualité des systèmes communautaires de santé à travers le pays. Une initiative communautaire et clinique intégrée, le renforcement des systèmes de santé et à base communautaire (RSSBC), vise à combler ces lacunes tout en renforçant le système sanitaire du secteur public dans le nord du Togo. Cette étude a pour objectif d’évaluer les effets et la stratégie de mise en œuvre de l’initiative de RSSBC sur 48 mois dans les zones desservies par 21 formations sanitaires du secteur public.

**Méthodes :** le modèle intégré de RSSBC comprend un ensemble d’interventions fondées sur des preuves ciblant des enfants âgés de moins de cinq ans, des femmes en âge de procréer et des personnes atteintes du VIH via (1) un engagement communautaire et de restitution ; (2) l’élimination des coûts dans les centres de santé pour la cible ; (3) des activités proactives de PCIME-C utilisant des agents de santé communautaires (ASCs) avec des services supplémentaires comprenant la planification familiale, des dépistages du VIH et le référencement des cas ; (4) un mentorat clinique et une supervision de soutien dans les formations sanitaires et (5) un renforcement de la chaîne d’approvisionnement et une amélioration d’un structure des formations sanitaires. À l’aide d’une étude pragmatique hybride de type II portant sur l’efficacité de la mise en œuvre, nous allons évaluer l’initiative de RSSBC par deux objectifs principaux : (1) déterminer l’efficacité à l’aide des variations des taux de mortalité chez les enfants âgés de moins de cinq ans et (2) évaluer la stratégie de mise en œuvre via des mesures de portée, d’adoption, de mise en œuvre et de maintien. Nous mènerons une évaluation combinant différentes méthodes en utilisant un cadre RE-AIM (portée, efficacité, adoption, mise en œuvre, maintien). Cette évaluation consiste en quatre éléments : (1) une étude randomisée en grappes avec permutation séquentielle (Stepped-wedge cluster randomized trial, SW-CRT*)* utilisant une enquête communautaire auprès des ménages, (2) des évaluations annuelles des formations sanitaires, (3) des entretiens avec les informateurs clés et (4) des évaluations du coût et du retour sur investissement pour chaque groupe randomisé.

**Discussion :** notre recherche a pour objectif de contribuer à l’amélioration constante de la qualité des initiatives, d’optimiser les facteurs de mise en œuvre, de fournir des connaissances en matière de prestation de services de santé et d’accélérer l’amélioration des systèmes sanitaires au Togo et de manière plus générale.

**Enregistrement de l’essai :** ClinicalTrials.gov Identifiant : NCT03694366, enregistré le 3 octobre 2018, [https : //clinicaltrials.gov/ct2/show/NCT03694366?term=NCT03694366&rank=1](https://clinicaltrials.gov/ct2/show/NCT03694366?term=NCT03694366&rank=1)

**Mots clés :** reproduction, maternel, santé infantile ; RE-AIM, systèmes de santé, agents de santé communautaires, supervision de soutien, PCIME, PCIME-C, Togo

**Contribution à la littérature**

- Ce protocole présente la manière d’utiliser les méthodologies scientifiques de la mise en œuvre pour conduire un essai pragmatique dans un contexte de faibles revenus de façon à améliorer la prestation de services sanitaires et à appliquer les conclusions dans la pratique.
- Il fournit un modèle de recherche sur la mise en œuvre intégrée dans le domaine des prestations de services du secteur public afin de faciliter la recherche fondée sur la demande et l’adoption des conclusions scientifiques dans la mise en œuvre de la politique. Une collaboration étroite et une appropriation parmi les praticiens, les décideurs politiques et les chercheurs sont cruciales afin d’aborder les questions de recherche pour un changement politique de valeur empirique dans les communautés locales.
- Cette étude évaluera l’efficacité et la stratégie de mise en œuvre d’une initiative communautaire et clinique intégrée dans le cadre d’un système de santé à faible revenu et vise à fournir des preuves aux décideurs politiques pouvant être généralisées afin d’aider à la prise de décisions nationales en matière de stratégie de santé communautaire.

**Contexte**

Tandis que le Togo a observé des réductions dans la mortalité infantile au cours des dernières décennies, une accélération du progrès est nécessaire pour atteindre l’Objectif de développement durable (ODD) 3.2 et réduire la mortalité néonatale à 12 pour 1 000 naissances vivantes et la mortalité des enfants âgés de moins de 5 ans à 25 pour 1 000 naissances vivantes (1,2). Les estimations régionales les plus récentes de 2013 et 2014 ont révélé que le taux de mortalité infantile dans la région de Kara, située dans le Nord, s’élève à 62 pour 1 000 naissances vivantes et que le taux de mortalité chez les enfants âgés de moins de 5 ans s’élève à 130 pour 1 000 naissances vivantes, par rapport aux taux nationaux de 49 et 88 pour 1 000 naissances vivantes, respectivement (3). La mortalité chez les enfants âgés de moins de cinq ans au Togo est due principalement à des maladies pour lesquelles il existe des interventions et traitements efficaces et de faible coût, telles que le paludisme, les infections aiguës des voies respiratoires inférieures et les maladies diarrhéiques (3). Bien que le Togo dispose d’un plan pour la Prise en Charge Intégrée des Maladies de l’Enfant (PCIME), d’une politique concernant la prise en charge intégrée des cas au niveau communautaire (PCIME-C), et d’un Programme Elargi de Vaccination (PEV), le pays n’a pas observé de réductions de la mortalité infantile comparables à celle des pays voisins homologues tels que le Burkina Faso, le Ghana et la Guinée(4). Par ailleurs, le Ministère de la Santé (MS) du Togo a rapporté que les taux nationaux d’utilisation des formations sanitaires du secteur public sont inférieurs à 30 %, bien que 62 % de la population vive dans un rayon de 5 km autour desdites formations sanitaires (5,6).

*Contexte et modèle intégré de renforcement des systèmes de santé et à base communautaire*

Santé Intégrée (SI) est une organisation non gouvernementale (ONG) travaillant en collaboration avec le MS togolais depuis 2004 ainsi qu’avec des organisations communautaires (OC). Ce partenariat public-privé se concentre sur l’intégration de services sanitaires à base communautaires et du secteur public dans la région de Kara, avec une focalisation initiale sur le VIH (7,8) puis une extension ultérieure aux soins primaires (9,10). Dans le cadre de la poursuite d’un objectif de recherche déterminant visant à améliorer les systèmes de santé à base communautaire, SI a formé le Community Health Systems Lab (CHSL), qui intègre la science de la mise en œuvre et la diffusion des résultats dans les opérations de SI, y compris le protocole d’étude actuel. Les détails organisationnels du CHSL sont présentés dans la figure 1. SI vise à favoriser une approche pédagogique du système sanitaire, via des preuves d’origine locale afin de renforcer l’efficacité de notre partenaire principal, le MS togolais, et de contribuer à la création de connaissances et à l’adoption par la diffusion aux collègues locaux, nationaux, et mondiaux.

**Figure 1**. Organigramme de la structure organisationnelle de Community Health Systems Lab (CHSL) au sein de Santé Intégrée

En 2014, SI a développé un nouveau partenariat avec le MS afin d’améliorer les services de santé primaires en se concentrant sur les causes majeures de mortalité prématurée dans les zones desservies par quatre centres de santé du secteur public dans la préfecture de Kozah, située dans le nord de la région de Kara. Ces interventions de santé à base communautaire, cliniques et préliminaires, sont désignées par l’expression « modèle intégré de renforcement des systèmes de santé à base communautaires (RSSBC) » (11). Ce modèle inclut un ensemble d’interventions fondées sur des preuves adaptées pour une mise en œuvre au Togo et est conforme aux recommandations internationales, y compris les directives aux agents de santé communautaires (ASC) émises par l’Organisation mondiale de la Santé (OMS) (12). La population cible comprend les enfants âgés de moins de cinq ans, les femmes enceintes ou en période post-partum, les femmes en âge de procréer pour des services de planification familiale et les personnes atteintes du VIH. Comme détaillé dans le tableau 1, l’ensemble RSSBC comprenant : (1) des réunions d’engagement communautaire et de restitution (13–15) ; (2) l’élimination des coûts au centre de santé pour la population cible (16–19) ; (3) les activités proactives de PCIME-C par le biais d’ASCs salariés avec des services supplémentaires étendus à la planification familiale et le conseil, des dépistages du VIH et le référencement des cas (11,19–27) ; (4) le mentorat clinique et la supervision de soutien dans les formations sanitaires (28–30) ; et (5) le renforcement de la chaîne logistique et l’amélioration des infrastructures de la formation sanitaire (15,31–33).

**Tableau 1** : Aperçu des éléments du modèle intégré de renforcement des systèmes santé et a base communautaires (RSSBC)

| **Élément du modèle RSSBC** | | **Détails** |
| --- | --- | --- |
| 1. | Réunions et restitutions sur l’engagement communautaire  *Preuve :* (13–15) | - Réunions de consultation pré-intervention avec les membres de la communauté. - Participation communautaire à la sélection des ASC. - Réunions d’examen communautaires semestrielles avec les dirigeants locaux, les membres de la communauté, le personnel des formations sanitaires et de SI. - Présentation des résultats de programmation et des mises à jour par le personnel de SI. - Commentaires de la communauté sur les défis de mise en œuvre, les succès et les éléments à améliorer concernant le RSSBC. |
| 2. | Élimination des coûts dans les formations sanitaires du secteur public mettant en œuvre l’intervention de SI  *Preuve :* (16–19) | - Comprend tous les enfants âgés de moins de cinq ans, les femmes enceintes ou venant d’accoucher, les femmes en âge de procréer pour des services de planification familiale et les personnes atteintes du VIH et qui viennent requérir des soins dans les centres d’étude. - Inclut les frais de consultation en formation sanitaire, les médicaments, les fournitures et les services fournis dans les centres d’intervention de SI et les recommandations de soins spécialisés vers les hôpitaux du district ou régionaux. - Frais sélectionnés et population couverte choisie en collaboration avec le MS et en conformité avec les recommandations nationales et mondiales. |
| 3. | PCIME-C proactive à l’aide d’ASCs équipés, supervisés et salariés, avec des services supplémentaires incluant la liaison vers la planification familiale et le conseil, le dépistage du VIH et les référencements de cas  *Preuve :* (11,19–27) | - Sélection des candidats dans la communauté par la direction locale, la formation sanitaire et le personnel de SI. - Préférence accordée aux résidents de sexe féminin qui répondent aux critères de sélection (certain niveau d’alphabétisation, résultats pré- et post-test, compétences connexes prouvées). - Formation de 23 jours en PCIME-C, santé maternelle et en conseil et dépistage du VIH, réalisée par le MS et le personnel de SI avant la prise de service ; - Formation de cinq jours sur le terrain dans le domaine de la planification familiale et du conseil par le MS et le personnel de SI. - Tous les supports de formation ont été élaborés en collaboration avec le MS, sont basés sur les directives nationales/internationales et sont fondés sur des données probantes fournies par l’Association Togolaise pour le Bien-Être Familial, Better Birth Project, Last Mile Health, Muso, Partners In Health. - Équipés en matériel (guides de formation, sac à dos, chronomètres, thermomètres, balances, dispositifs de mesure du tour de bras, tests rapides, traitement médical pour les cas basiques de PCIME-C, cahiers, stylos). - Consultations ASCs, recommandations, médicaments et matériel fournis gratuitement. - Supervision d’assistance avec parrainage et encadrement par des superviseurs de SI (infirmiers/assistants médicaux). - Contrôle régulier des prestations fournies par les ASCs, effectué à l’aide des données de programmation régulières et des commentaires de la communauté. - Les ASC reçoivent un salaire régulier et équitable pour leur travail à temps plein via la recherche proactive de cas et le suivi. |
| 4. | Mentorat clinique et supervision soutien par un superviseur de pairs formé dans des formations sanitaires du secteur public.  *Preuve :* (28–30) | - Formation de quatre jours sur le terrain avant la prise de service dans le domaine de la santé maternelle, sexuelle, néonatale et infantile, ainsi qu’en lien avec le VIH, réalisée par un mentorat clinique (infirmier/assistant médical) et un directeur médical (médecin). - Supports de formation élaborés en collaboration avec le MS, basés sur les directives nationales/internationales et fondés sur des données probantes fournies par l’OMS, l’American Academy of Pediatrics, Ariadne Labs, Better Birth Project, Last Mile Health, Partners In Health, Muso. - Supervision d’assistance hebdomadaire en formation sanitaire par les formateurs-cliniciens de SI (personnel infirmier, sage-femme, assistants médicaux) avec une expérience antérieure dans les formations sanitaires du secteur public. - Contrôle régulier des prestations fournies par le personnel de la formation sanitaire, effectué à l’aide des données de programmation régulières et des commentaires de la communauté. |
| 5. | Améliorations basiques de l’infrastructure et des équipements, et formation à la gestion de la chaîne d’approvisionnement et des gestionnaires de la pharmacie  *Preuve :* (15,31–33) | - Évaluation formelle de l’infrastructure et des besoins en équipements réalisée avec le MS à l’aide de l’outil SARA de l’OMS(34). - Favorise les améliorations structurelles afin de rénover les formations sanitaires. - Équipe la formation sanitaire en médicaments et matériel essentiels identifiés par une évaluation et des protocoles sanitaires nationaux. - Formation sur le terrain aux pratiques de gestion de la chaîne d’approvisionnement, y compris la conservation appropriée des médicaments, le remplissage des fiches de stock et les commandes en fonction de la consommation mensuelle d’intrants. - Supervision et assistance régulières par le mentorat clinique de SI. |

Les résultats préliminaires de l’essai pilote de mise en œuvre du RSSBC dans la préfecture de Kozah ont révélé une réduction de la mortalité chez les enfants âgés de moins de cinq ans et une augmentation de l’utilisation des services de santé pour les services sanitaires destinés aux enfants et à leurs mères sur l’ensemble des quatre sites d’intervention (ClinicalTrials.gov Identifiant : NCT03773913). Cette évaluation a été menée à l’aide d’une enquête auprès des ménages répétée, représentative de la population et transversale. Nous anticipons des résultats à 48 mois de la mise en œuvre en 2019.

Après cette phase pilote préliminaire réussie et en tenant compte des besoins, le MS a demandé à SI d’étendre le modèle de RSSBC à des sites supplémentaires à compter de 2018. En collaboration avec le MS et des partenaires techniques, SI reproduit le modèle RSSBC dans 21 formations sanitaires ruraux distincts, situés dans 4 préfectures supplémentaires. En collaboration avec les partenaires du MS, l’équipe du programme de RSSBC a conçu une stratégie de mise en œuvre pour cette extension afin de favoriser l’adoption, la mise en œuvre et la durabilité du modèle (35–37). Cette stratégie permet un déploiement vers une nouvelle préfecture tous les 12 mois et se fonde sur l’expérience pilote, les facteurs contextuels locaux ainsi que sur des considérations d’ordre budgétaire et de faisabilité. En tant que membre de cette extension lancée en 2018, SI et les partenaires du MS ont conçu un essai randomisé en grappes avec permutation séquentielle pour permettre une évaluation rigoureuse de l’efficacité de l’intervention et des stratégies de mise en œuvre destiné à transmettre des informations nécessaires à la politique nationale.

*Justification de la conception de l’étude*

Nous avons sélectionné une étude hybride pragmatique de type II d’efficacité et de mise en œuvre, puisqu’elle permet des évaluations simultanées combinant différentes méthodes portant sur l’efficacité de l’intervention et sur les stratégies de mise en œuvre dans le cadre de systèmes sanitaires « en situation réelle » (36,38). Via cette conception convergente (39), nous espérons diffuser régulièrement (40) nos résultats d’étude aux partenaires du MS. Les mesures de l’efficacité fourniront des preuves soutenant ou non l’impact de l’intervention de RSSBC sur la mortalité infantile et, via cette conception pragmatique, fourniront des estimations plus propices à la généralisation par rapport aux conceptions d’études traditionnelles (36). Évaluer la stratégie de mise en œuvre créera des connaissances sur les résultats du processus et la faisabilité, incluant les obstacles et les facteurs favorables, les éléments principaux qui peuvent être généralisés et où l’adaptation locale est indispensable pour une reproduction dans d’autres situations. Ces connaissances pouvant être mises en pratique sont indispensables pour les acteurs de la mise en œuvre dans les formations sanitaires à faible et moyen revenu, en particulier pour les interventions de systèmes sanitaires complexes (12,41).

Bien que notre étude pilote préliminaire à Kozah ait fourni des données initiales suggérant l’efficacité du RSSBC, ces résultats présentent une capacité limitée dans l’établissement d’une causalité en raison d’une conception à bras unique et de l’absence de groupe de comparaison valide. En outre, notre étude pilote n’a pas été conçue pour évaluer les stratégies de mise en œuvre requises afin de fournir des données pouvant être généralisées et ainsi aider à la reproduction et à la détermination de l’échelle.

Afin de remédier à ces limitations et évaluer rigoureusement l’efficacité du RSSBC et la stratégie de mise en œuvre, nous conduirons une évaluation sommative combinant différentes méthodes à l’aide d’un cadre RE-AIM (portée, efficacité, adoption, mise en œuvre, maintien) modifié (42). Ce cadre est un outil pratique qui permet d’évaluer les interventions complexes dans la pratique réelle, y compris les mesures de l’efficacité clinique et la stratégie de mise en œuvre (43). L’efficacité sera mesurée à l’aide du résultat principal, la mortalité chez les enfants âgés de moins de cinq ans, au moyen d’une conception contrôlée randomisée par groupes avec permutations. La stratégie de mise en œuvre sera évaluée par les mesures de portée, d’adoption, de mise en œuvre et de maintien de l’intervention de RSSBC via le recueil de données auprès des informateurs clés, des formations sanitaires et des populations générales desservies (44). Ce cadre d’évaluation adapté permettra d’améliorer la conception traditionnelle avec permutations en incluant les mesures de la fidélité de la mise en œuvre (45).

Compte tenu des besoins initiaux de la population, des résultats d’efficacité préliminaires et de la requête du MS pour un passage à l’échelle, nous avons jugé non éthique de suspendre l’intervention dans l’un des sites de comparaison. Les contraintes logistiques et financières pour le lancement du modèle de RSSBC imposaient déjà la mise en œuvre séquentielle nécessaire à un essai avec grappes échelonnées.

Ce protocole est la meilleure solution compte tenu des limitations du pilote initial suivant une conception à bras unique, répétée et transversale et du besoin de conclusions pouvant être généralisées afin de soutenir la transposition des résultats dans la pratique (45,46). Le recueil annuel des données, parallèlement à l’augmentation de la demande en ressources et des coûts, a été jugé réalisable et bénéfique, puisqu’il permettra une analyse quantitative et qualitative régulière des données et une diffusion itérative des résultats, en interne pour soutenir les améliorations apportées au programme et avec les partenaires du MS pour des pratiques de recherche intégrées (47). Notre objectif dans ce document est de décrire le protocole de recherche de cette étude hybride pragmatique d’efficacité et de mise en œuvre visant à mesurer l’efficacité et la stratégie de mise en œuvre de l’initiative de RSSBC sur 48 mois dans le nord du Togo rural en utilisant un cadre RE-AIM.

**Méthodes/Conception**

**Objectifs de l’étude**

L’objectif de cette étude est d’optimiser la mise en œuvre du modèle de RSSBC à l’aide du cadre d’évaluation RE-AIM adapté. À cette fin, nous poursuivons deux objectifs principaux : (1) déterminer l’efficacité du modèle de RSSBC et (2) évaluer la stratégie de mise en œuvre via des mesures de portée, d’adoption, de mise en œuvre et de maintien. Nos objectifs spécifiques d’étude incluent les éléments suivants :

Objectif principal 1 : efficacité

(1) Analyser les variations longitudinales dans la mortalité et la morbidité maternelles et infantiles, la qualité des paramètres de soin ainsi que la préparation des formations sanitaires du secteur public dans les zones desservies.

Objectif principal 2 : stratégie de mise en œuvre

(1) Identifier les obstacles et les facilitateurs contribuant à l’accès et à la qualité des services de RSSBC ;

(2) Mesurer les variations dans la couverture, les taux d’utilisation des services sanitaires et l’adoption de l’intervention ;

(3) Déterminer les coûts de mise en œuvre du RSSBC et les estimations de retour sur investissement.

**Conception**

Cette étude utilise une conception hybride pragmatique de type II de l’efficacité et de la mise en œuvre (36) afin d’évaluer les deux objectifs principaux d’efficacité et de stratégie de mise en œuvre de l’initiative de RSSBC à l’aide d’un cadre scientifique de mise en œuvre RE-AIM(42). La liste de contrôle CONSORT figure dans le document supplémentaire 1. Nous inclurons quatre éléments d’étude distincts : (1) une étude randomisée en grappes (cluster) avec permutation séquentielle (*Stepped-wedge cluster randomized trial, SW-CRT*) utilisant une enquête communautaire auprès des ménages, (2) des évaluations annuelles des formations sanitaires, (3) des entretiens avec les informateurs clés menés un an après la réception post-intervention et (4) des analyses du coût et du retour sur investissement annuelles réalisées à l’aide de l’Outil de santé communautaire et de calcul des coûts (48) et de l’Outil de calcul des vies sauvées *(Lives Saved Tool, LiST*) (49). Vous trouverez plus de détails sur chaque élément de l’étude dans le tableau 2 ci-dessous qui reprend le calendrier.

**Tableau 2** : Recueil des données et calendrier de l’initiation du renforcement des systèmes communautaires de santé intégrée (RSSBC) basé sur une mise en place échelonnée*

| **Groupe** | **Activité**** | **Mois**  **1 à 2** | **Mois**  **3 à 12** | **Mois**  **13 à 14** | **Mois**  **15 à 24** | **Mois**  **25 à 26** | **Mois**  **27 à 36** | **Mois**  **37 à 38** | **Mois**  **39 à 48** | **Mois**  **49 à 50** |
| --- | --- | --- | --- | --- | --- | --- | --- | --- | --- | --- |
|  |  | *Mai/Juin*  *2018* | *Juillet  2018 à avril 2019* | *Mai/Juin*  *2019* | *Juillet  2019 à avril 2020* | *Mai/Juin*  *2020* | *Juillet  2020 à avril 2021* | *Mai/Juin 2021* | *Juillet  2021 à avril 2022* | *Mai/Juin  2022* |
| Préfecture 1 | Lancement RSSBC | **X** | **X** | **X** | **X** | **X** | **X** | **X** | **X** | **X** |
|  | Enquête CM | **X** |  | **X** |  | **X** |  | **X** |  | **X** |
|  | Enquête FSs | **X** |  | **X** |  | **X** |  | **X** |  | **X** |
|  | IC |  | **X** |  | **X** |  | **X** |  | **X** |  |
|  | Enquête coût |  | **X** |  | **X** |  | **X** |  | **X** |  |
| Préfecture 2 | Lancement RSSBC |  |  | **X** | **X** | **X** | **X** | **X** | **X** | **X** |
|  | Enquête CM | **X** |  | **X** |  | **X** |  | **X** |  | **X** |
|  | Enquête FSs | **X** |  | **X** |  | **X** |  | **X** |  | **X** |
|  | IC |  |  |  | **X** |  | **X** |  | **X** |  |
|  | Enquête coût |  |  |  | **X** |  | **X** |  | **X** |  |
| Préfecture 3 | Lancement RSSBC |  |  |  |  | **X** | **X** | **X** | **X** | **X** |
|  | Enquête CM | **X** |  | **X** |  | **X** |  | **X** |  | **X** |
|  | Enquête FSs | **X** |  | **X** |  | **X** |  | **X** |  | **X** |
|  | IC |  |  |  |  |  | **X** |  | **X** |  |
|  | Enquête coût |  |  |  |  |  | **X** |  | **X** |  |
| Préfecture 4 | Lancement RSSBC |  |  |  |  |  |  | **X** | **X** | **X** |
|  | Enquête CM | **X** |  | **X** |  | **X** |  | **X** |  | **X** |
|  | Enquête FSs | **X** |  | **X** |  | **X** |  | **X** |  | **X** |
|  | IC |  |  |  |  |  |  |  | **X** | **X** |
|  | Enquête coût |  |  |  |  |  |  |  | **X** |  |

* Conforme au diagramme d’extension CONSORT pour *le essai aléatoire pragmatique et contrôle par grappes échelonnées (Stepped-wedge cluster randomized trial, SW-CRT)*) (50)

***Enquête CM* enquête communautaire auprès des ménages, *Enquêtes FSs* évaluations des formations sanitaires, *IC* entretiens avec les informateurs clés, *Enquête de coût* évaluation du coût et du retour sur investissement

*Etude randomisée en grappes avec permutation séquentielle (Stepped-wedge cluster randomized trial, SW-CRT) :* cette conception pragmatique optimise le déploiement séquentiel ou échelonné du modèle de RSSBC et rendra possible une évaluation de l’efficacité et de la stratégie de mise en œuvre via des mesures de portée et d’adoption en comparant les groupes organisés géographiquement (51,52) Elle comprend quatre groupes organisant l’intervention dans les formations sanitaires par préfecture : Bassar, Binah, Dankpen et Kéran. L’ordre d’initiation du RSSBC dans les groupes sera randomisé annuellement en quatre étapes, puisqu’il a été déterminé indépendamment par l’équipe des programmes de SI que les besoins initiaux en matière de santé étaient similaires pour chaque groupe. Des enquêtes communautaires menées auprès des ménages et adaptées des modules d’Enquête démographique et sanitaire (EDS) mis en place précédemment au Togo(3) et se concentrant sur les données démographiques, de santé maternelle et infantile seront menées dans chaque groupe à l’inclusion puis à 12, 24, 36 et 48 mois.

*Évaluations de la formation sanitaire :* ces évaluations emploieront des enquêtes de la formation sanitaire basées sur l’outil d’Évaluation de la préparation et de la disponibilité des services (Service Availability and Readiness Assessment tool, SARA) de l’OMS(34) et fourniront des informations sur l’efficacité en rapport avec la qualité des services au niveau de la formation sanitaire. Ces enquêtes seront menées auprès de chaque formation sanitaire annuellement, à l’inclusion, puis à 12, 24, 36 et 48 mois.

*Entretiens menés avec les informateurs clés :* des entretiens qualitatifs seront réalisés avec les informateurs clés afin d’évaluer les obstacles et les facilitateurs en rapport avec la fidélité et la faisabilité de la mise en œuvre du programme tout en documentant également les facteurs contextuels. Les premiers entretiens avec les informateurs clés seront menés 12 mois après le lancement de l’intervention puis à des intervalles de 12 mois jusqu’à la fin de l’étude pour chaque groupe.

*Évaluation du coût et du retour sur investissement :* les coûts et retours sur investissement du programme RSSBC seront mesurés à l’aide de l’Outil de planification de la santé communautaire et de calcul des coûts (48) et de l’Outil de calcul des vies sauvées (49) afin d’évaluer les approches stratégiques de mise en œuvre et de guider les délibérations sur le maintien et sur les efforts de planification nationaux. La première évaluation sera menée 12 mois après le lancement de l’intervention puis à des intervalles de 12 mois jusqu’à la fin de l’étude pour chaque groupe.

**Conditions de l’étude**

L’étude sera menée dans les zones desservies par 21 formations sanitaires du secteur public dans les préfectures rurales de la région de Kara : Bassar, Binah, Dankpen et Kéran. La population totale s’élève à environ 237 807 personnes. Les centres de l’étude ont été sélectionnés par les partenaires du MS et le personnel de SI chargé de la programmation en fonction des besoins sanitaires perçus au niveau de la population, des activités liées aux programmes régionaux publics-privés en cours et de la taille de la population. Tous les centres sélectionnés sont des formations sanitaires de soins primaires gérés par le MS(53) et destinés aux populations rurales. La population desservie et les taux d’utilisation estimés pour ces centres sont listés par préfecture dans le tableau 3. Comme décrit dans le tableau 2, l’initiative de RSSBC sera mise en œuvre par préfecture de manière séquentielle, chaque année, au sein des 21 centres présélectionnés.

**Tableau 3** : Liste des formations sanitaires d’étude (*N =*21) avec la population desservie estimée de référence (*N =*181 111) et les taux d’utilisation des formations sanitaires

| **Préfecture** | **Formation sanitaire** | **Population desservie*** | **Taux d’utilisation de la formation sanitaire**** |
| --- | --- | --- | --- |
| Bassar | Bangéli | 16 169 | 42 % |
| Bassar | Kabou-Sara | 10 054 | 56 % |
| Bassar | Koundoum | 7 428 | 37 % |
| Bassar | Manga | 5 006 | 28 % |
| Bassar | Sanda-Afohou | 5 514 | 43 % |
| Binah | Asseré | 4 446 | 19 % |
| Binah | Boufalé | 4 212 | 48 % |
| Binah | Kouyorira | 4 364 | 26 % |
| Binah | N’Djei | 3 258 | 53 % |
| Binah | Pessaré | 8 002 | 26 % |
| Binah | Sirka | 5 980 | 51 % |
| Binah | Solla | 5 960 | 58 % |
| Dankpen | Koutière | 13 097 | 12 % |
| Dankpen | Kpétab | 8 208 | 22 % |
| Dankpen | Naware | 19 531 | 10 % |
| Dankpen | Solidarité | 8 157 | 24 % |
| Kéran | Kokou-Temberma | 8 722 | Inconnu |
| Kéran | Nadoba | 16 593 | Inconnu |
| Kéran | Natiponi | 7 636 | Inconnu |
| Kéran | Pangouda | 10 735 | Inconnu |
| Kéran | Warengo | 8 039 | Inconnu |

* Données provenant des estimations démographiques du ministère de la Santé de 2016 et de l’échantillonnage populationnel de référence de Santé Intégrée de 2018.

** Données issues des rapports annuels sur les préfectures émis par le ministère de la Santé.

**Critères d’inclusion**

L’éligibilité pour l’inclusion est décrite ci-dessous en fonction de l’élément à l’étude pour les enquêtes communautaires auprès des ménages, les enquêtes des formations sanitaires et les entretiens qualitatifs.

*Enquêtes communautaires auprès des ménages :* les femmes âgées de 15 à 49 ans résidant dans un foyer sélectionné au sein de la zone desservie sont incluses. Les participantes âgées de 15 à 17 ans ne seront incluses que si elles ont été ou sont actuellement enceintes et si elles disposent d’une autorisation parentale. Les ménages seront sélectionnés de manière aléatoire chaque année à l’aide d’une méthodologie d’échantillonnage systématique pondérée en fonction de la population. S’il y a plus d’une répondante éligible dans le foyer, une seule sera sélectionnée de manière aléatoire selon la grille de sélection de Kish(54). Toutes les participantes auront la possibilité de refuser de participer au cours du processus de consentement éclairé.

*Enquêtes auprès des formations sanitaires :* l’ensemble des 21 formations sanitaires seront interrogées. Les informations concernant les formations sanitaires figurent dans le tableau 3.

*Entretiens menés avec les informateurs clés :* cet élément inclura les individus âgés de 18 ans ou plus qui sont soit des partenaires de mise en œuvre soit du personnel d’intervention dans la formation sanitaire. Les partenaires de mise en œuvre sont définis comme les membres du personnel de programmation de SI employés dans la zone d’intérêt desservie. Les membres du personnel de la formation sanitaire sont définis comme les employés cliniques ou administratifs du MS dans l’un des 21 centres sanitaires. Environ 42 informateurs clés issus des 21 formations sanitaires (21 partenaires de mise en œuvre et 21 prestataires) seront inclus.

**Détermination de la taille de l’échantillon**

L’étude dispose d’une puissance suffisante pour détecter une variation par groupe dans la mortalité des enfants âgés de moins de 5 ans pour 1 000 naissances vivantes. Une taille d’échantillon comprenant 7 600 participantes fournira une puissance de 80 % permettant de détecter une réduction estimée à 30 % ou plus de la mortalité chez les enfants âgés de moins de 5 ans par rapport à la référence estimée à 70 pour 1 000 naissances vivantes, avec un alpha de 0,05, une corrélation au sein du groupe de 0,005, un taux de non-répondeurs de 20 % et une estimation de 0,5 enfant âgé de moins de 5 ans par participante (9,55). L’ampleur de l’effet est une estimation prudente basée sur l’expérience pilote précédente (ClinicalTrials.gov Identifiant : NCT03773913).

*Randomisation*

L’ordre du groupe pour la mise en œuvre de chaque étape sera déterminé de manière aléatoire par un conseiller technique externe à l’aide d’un générateur de nombres aléatoires. La randomisation sera réalisée chaque année, huit mois avant le déploiement de l’intervention dans le groupe suivant. Cela permettra de mettre en aveugle l’ordre aléatoire des groupes pour le personnel de SI et du MS impliqué dans la mise en œuvre, tout en permettant également de réaliser l’étape de planification annuelle sur huit mois avant le lancement de l’intervention. Chaque groupe représente une préfecture, avec un total de 21 formations sanitaires présélectionnés à travers chacune des quatre préfectures.

**Recueil et analyse des données**

Toutes les mesures seront organisées à l’aide d’un cadre RE-AIM (portée, efficacité, adoption, mise en œuvre et maintien) modifié (56). Le tableau 4 résume les plans concernant le recueil des données et l’analyse organisés selon l’objectif principal et les domaines RE-AIM adaptés.

**Tableau 4** : Résumé des mesures clés de l’étude organisé par domaine à l’aide du cadre d’évaluation RE-AIM

| **Objectif principal** | **Domaine du cadre RE-AIM** | **Résultat(s)/Indicateur(s)** | **Source des données** | **Définition/Explication de l’indicateur** |
| --- | --- | --- | --- | --- |
| *Objectif principal 2 : stratégie de mise en œuvre* | Portée | *Couverture du service sanitaire* | | |
|  |  | Visites à domicile des ASC | Enquête communautaire auprès des ménages | Proportion de participants rapportant une visite à domicile d’un ASC de SI au cours de l’année précédente. |
|  |  | Traitement à domicile administré par un ASC | Enquête communautaire auprès des ménages | Proportion de participants rapportant un traitement à domicile administré par un ASC de SI au cours de l’année précédente. |
|  |  | Traitement au centre de santé | Enquête communautaire auprès des ménages | Proportion de participants rapportant des soins reçus dans un formation sanitaire au cours de l’année précédente. |
|  |  | *Rapidité d’accès aux services destinés à la santé infantile* | | |
|  |  | Couverture du paludisme | Enquête communautaire auprès des ménages | Proportion d’enfants âgés de moins de cinq ans rapportés comme étant fébriles et recevant un traitement conforme aux directives dans les 24 heures suivant l’apparition des symptômes. |
|  |  | Couverture de la pneumonie | Enquête communautaire auprès des ménages | Proportion d’enfants âgés de moins de cinq ans présentant une toux et proportion de ceux recevant un traitement conforme aux directives dans les 24 heures suivant l’apparition des symptômes. |
|  |  | Couverture des maladies gastro-intestinales | Enquête communautaire auprès des ménages | Proportion d’enfants âgés de moins de cinq ans présentant une diarrhée et recevant un traitement conforme aux directives dans les 24 heures suivant l’apparition des symptômes. |
|  |  | Couverture de la malnutrition | Enquête communautaire auprès des ménages | Proportion d’enfants âgés de moins de cinq ans présentant une malnutrition et recevant un traitement efficace. |
|  |  | Estimation de couverture en ce qui concerne les soins prénataux | Enquête communautaire auprès des ménages | Proportion de femmes enceintes ayant assisté à quatre visites de soins prénataux au cours des deux dernières années. |
|  |  | Pourcentage de naissance dans une formation sanitaire | Enquête communautaire auprès des ménages | Proportion de femmes enceintes ayant accouché dans une formation sanitaire au cours des deux dernières années. |
|  |  | Pourcentage de naissances à domicile | Enquête communautaire auprès des ménages | Proportion de femmes enceintes ayant accouché à domicile au cours des deux dernières années. |
|  |  | Estimation de couverture en ce qui concerne les soins postnataux | Enquête communautaire auprès des ménages | Proportion de femmes en période postpartum ayant reçu des soins postnataux au cours des deux dernières années. |
| *Objectif principal 1 : efficacité* | Efficacité | *Résultat principal* | | |
|  |  | Taux de mortalité infanto-juvenile | Enquête communautaire auprès des ménages | À l’aide d’un tableau standard rapportant les antécédents de naissance/décès, calcul des taux de mortalité chez les enfants âgés de moins de cinq ans et comparaison du risque de décès avant l’âge de cinq ans sur l’ensemble des enquêtes à l’aide du modèle de régression à risque proportionnel de Cox en prenant l’année de l’enquête comme variable explicative. Les enfants encore en vie et âgés de moins de cinq ans au moment de l’enquête seront censurés à droite. |
|  |  | *Résultats secondaires* | | |
|  |  | Taux de mortalité néonatale | Enquête communautaire auprès des ménages | Les taux de mortalité néonatale à partir de toutes les naissances rapportées par les répondantes au cours des 5 années précédant l’enquête et à l’aide des mêmes méthodes que celles décrites pour la mortalité chez les enfants âgés de moins de cinq ans, ajustées à 28 jours. |
|  |  | Taux de mortalité des enfants âgés de moins d’un an | Enquête communautaire auprès des ménages | Les taux de mortalité chez les enfants âgés de moins d’un an à partir de toutes les naissances rapportées par les répondantes au cours des cinq années précédant l’enquête et à l’aide des mêmes méthodes que celles décrites pour la mortalité chez les enfants âgés de moins de cinq ans, ajustées à un an. |
|  |  | Taux de mortalité des enfants âgés de moins de deux ans | Enquête communautaire auprès des ménages | Les taux de mortalité chez les enfants âgés de moins de deux ans à partir de toutes les naissances rapportées par les répondantes au cours des cinq années précédant l’enquête et à l’aide des mêmes méthodes que celles décrites pour la mortalité chez les enfants âgés de moins de cinq ans, ajustées à deux ans. |
|  |  | Taux de mortalité maternelle | Enquête communautaire auprès des ménages | Analyse de la mortalité maternelle exploratoire basée sur les rapports de la sororité (57,58). |
|  |  | *Qualité des paramètres de soin* | | |
|  |  | Rapidité/Promptitude des soins pédiatriques pour le paludisme | Données de programmation régulières | Proportion d’enfants âgés de moins de 5 ans rapportés comme étant fébriles et proportion de ceux ayant reçu un traitement efficace contre le paludisme dans les 24 heures suivant l’apparition des symptômes. |
|  |  | Rapidité/Promptitude des soins pédiatriques pour la pneumonie | Données de programmation régulières | Proportion d’enfants âgés de moins de 5 ans rapportés comme présentant une toux et proportion de ceux ayant reçu un traitement efficace contre la pneumonie dans les 24 heures suivant l’apparition des symptômes. |
|  |  | Rapidité/Promptitude des soins pédiatriques pour la diarrhée | Données de programmation régulières | Proportion d’enfants âgés de moins de 5 ans rapportés comme présentant une diarrhée et proportion de ceux ayant reçu un traitement efficace contre la diarrhée dans les 24 heures suivant l’apparition des symptômes. |
|  |  | Compétence technique des ASC | Données de programmation régulières | Proportion d’ASC de SI adhérant aux protocoles basés sur des données probantes pour la PCIME-C et la santé maternelle. |
|  |  | Compétences techniques du personnel clinique des formations sanitaires | Données de programmation régulières | Proportion de personnel clinique des centres adhérant aux protocoles basés sur des données probantes pour la PCIME-C et la santé maternelle. |
|  |  | Équité | Enquête communautaire auprès des ménages | Différences d’accès concernant la mortalité infantile entre les quintiles de richesse maternelle, la distance jusqu’à la formation sanitaire et le niveau d’éducation. |
|  |  | Score de préparation aux soins | Évaluations de la formation sanitaire | Examen des changements opérés dans la formation sanitaire en rapport avec l’approvisionnement, l’infrastructure physique et la gestion via le score de préparation annuel (34). |
| *Objectif principal 2 : stratégie de mise en œuvre* | Adoption | *Engagement communautaire* | | |
|  |  | Sessions d’engagement communautaire | Données de programmation régulières | Nombre de forums communautaires et de membres de la communauté présents. |
|  |  | *Changement de comportement au niveau du participant individuel* | | |
|  |  | Cascades de soins pédiatriques pour la fièvre, la pneumonie et la diarrhée | Enquête communautaire auprès des ménages | Changements dans le temps au niveau du comportement lié à la recherche de soins, pour la fièvre, la pneumonie et la diarrhée chez les patients se présentant à des cliniques, à des ASC ou à des centres non cliniques. Test destiné à vérifier si ces proportions augmentent à l’aide de la même approche que les modèles linéaires généralisés à effets mixtes, comme décrit dans la mesure du résultat principal d’efficacité. |
|  |  | Cascade des soins prénataux, des accouchements en formation sanitaire et des soins postnatals pour les femmes en âge d’avoir des enfants. | Enquête communautaire auprès des ménages | Changement dans le temps du comportement lié à la recherche de soins chez les femmes enceintes ; prestations fournies par les ASC de SI, accouchement en formation sanitaire, ainsi que suivi de soins anténataux et postnataux. Test destiné à vérifier si ces proportions augmentent à l’aide de la même approche que les modèles linéaires généralisés à effets mixtes, comme décrit dans la mesure du résultat principal d’efficacité. |
| *Objectif principal 2 : stratégie de mise en œuvre* | Mise en œuvre | *Entretiens qualitatifs* | | |
|  |  | Fidélité | Entretiens menés auprès des informateurs clés | Degré de confirmation que la ou les interventions ont été mises en œuvre comme prévu dans le protocole original. |
|  |  | Faisabilité | Entretiens menés auprès des informateurs clés | Degré de confirmation que l’intervention a pu être menée dans un contexte particulier. |
|  |  | Contexte extérieur (59) | Entretiens menés auprès des informateurs clés | Macrofacteurs externes, y compris d’ordre social, liés au financement et à la direction. |
|  |  | Contexte interne (59) | Entretiens menés auprès des informateurs clés | Microfacteurs internes, y compris ceux liés au partenariat entre SI/MS, aux différents problèmes concernant les rôles de SI et du MS, aux retours, aux formations sanitaires, à la communauté, aux ménages et aux individus. |
| *Objectif principal 2 : stratégie de mise en œuvre* | Maintien | *Évaluation des coûts et du retour sur investissement* | | |
|  |  | Coût annuel par personne | Enquêtes sur les coûts | Coût par personne comparé au financement actuel du MS à l’aide de l’Outil de planification de la santé communautaire et de calcul des coûts (48), au niveau du groupe. |
|  |  | Retour sur investissement | Enquêtes sur les coûts | Retour sur investissement calculé à l’aide de l’Outil de planification de la santé communautaire et de calcul des coûts (48) et de l’Outil de calcul des vies sauvées (49), avec le résultat principal au niveau du groupe. |

*Objectif principal 1 : efficacité*

*Efficacité* : Nous définissons les mesures d’*efficacité* comme celles évaluant l’impact de l’initiative de RSSBC à l’aide d’enquêtes communautaires annuelles auprès des ménages, des données de programmation régulières et des évaluations des formations sanitaires. Le résultat primaire au niveau communautaire par préfecture utilise les taux de mortalité chez les enfants âgés de moins de cinq ans, ainsi que les résultats secondaires concernant les taux de mortalité chez la mère et chez les nouveau-nés, les nourrissons âgés de moins d’un an ou de moins de deux ans. Nous évaluerons en outre la qualité des paramètres de soin en nous focalisant sur la rapidité des soins prodigués aux enfants via la promptitude de l’administration du traitement après le diagnostic d’une maladie et via les scores de préparation de formation sanitaire. Enfin, nous évaluerons l’équité à l’aide d’analyses secondaires de la mortalité chez les enfants âgés de moins de cinq ans par quintiles de richesse des ménages, niveaux d’éducation maternelle et distance jusqu’à la formation sanitaire la plus proche.

Résultat primaire : taux de mortalité chez les enfants âgés de moins de cinq ans : nous calculerons les taux de mortalité chez les enfants âgés de moins de cinq ans en fonction de toutes les naissances rapportées par les répondantes à l’aide d’un tableau standard rapportant les antécédents de naissance/décès. Nous calculerons les taux de mortalité chez les enfants âgés de moins de cinq ans et comparerons le risque de décès avant l’âge de cinq ans sur l’ensemble des enquêtes à l’aide du modèle de régression à risque proportionnel de Cox en prenant l’exposition à l’intervention comme variable explicative. Les enfants encore en vie et âgés de moins de cinq ans au moment de l’enquête seront censurés à droite.

Résultats secondaires : taux de mortalité chez les nouveau-nés, les nourrissons âgés de moins d’un an et de moins de deux ans : nous calculerons les taux de mortalité chez les nouveau-nés, les nourrissons âgés de moins d’un an et de moins de deux ans en fonction de toutes les naissances rapportées par les répondantes en utilisant les mêmes méthodes que celles décrites ci-dessus, ajustées pour 28 jours, 1 an et 2 ans.

Résultat secondaire : mortalité maternelle : nous élaborerons une analyse de la mortalité maternelle exploratoire basée sur les rapports de la sororité (57,58).

*Objectif principal 2 : stratégie de mise en œuvre* :

*Portée* : nous définissons les mesures de *portée* comme la proportion de population cible ayant obtenu l’accès aux services de l’initiative de RSSBC à l’aide des enquêtes communautaires annuelles menées auprès des ménages. Nous évaluerons la stratégie de mise en œuvre via la participation individuelle au RSSBC grâce à l’utilisation des services sanitaires au niveau communautaire (vis-à-vis des ASCs) et au niveau de la formation sanitaire, en utilisant des estimations de couverture des services sanitaires et la promptitude de l’accès aux services pour la santé infantile. Nous utiliserons un modèle linéaire généralisé à effets mixtes pour comparer les proportions pré intervention et post intervention pour chaque indicateur de *portée* tout en ajustant en fonction du groupe au niveau de la formation sanitaire et du district, en fonction du temps, et en permettant que les estimations au niveau du district soient des effets aléatoires. Notre analyse primaire n’inclura pas un ajustement en fonction des caractéristiques individuelles, puisque chaque préfecture sera son propre témoin.

*Adoption* : nous définissons les mesures d’*adoption* pour cette étude comme la proportion de la communauté et de prestataires transformant les comportements liés à la recherche ou à l’administration de soins de santé. Notre évaluation de l’*adoption* sera réalisée à l’aide des données de programmation régulières et des enquêtes communautaires annuelles menées auprès des ménages. Via ce domaine, nous évaluerons les mesures de la stratégie de mise en œuvre en utilisant l’engagement au niveau communautaire et le changement comportemental par l’adoption au niveau individuel de l’intervention de RSSBC. Nous évaluerons si ces proportions augmentent, en appliquant la même approche que celle décrite dans les mesures d’évaluation de la *portée* via des modèles linéaires généralisés à effets mixtes.

*Mise en œuvre* : les mesures de la *mise en œuvre* sont exprimées en fidélité et faisabilité ainsi qu’en documentant les facteurs contextuels. L’évaluation de la *mise en œuvre* sera réalisée à l’aide des évaluations qualitatives des informateurs clés (lors des entretiens approfondis avec les partenaires de mise en œuvre et le personnel de la formation sanitaire) qui seront menées 1 an après l’intervention au niveau du groupe (préfecture). Cela complètera les données quantitatives recueillies afin d’évaluer la stratégie de mise en œuvre et permettra d’évaluer les thèmes émergeants.

*Maintien :* notre étude définit les mesures de *maintien* comme les coûts requis pour fournir et maintenir le modèle de RSSBC. L’évaluation du *maintien* sera réalisée au moyen de l’Analyse du coût et du retour sur investissement, qui sera menée chaque année de la mise en œuvre au niveau du groupe (préfecture). Cette analyse évaluera les coûts de mise en œuvre du programme en fonction de la conception de la stratégie en utilisant rétrospectivement l’Outil de planification de la santé communautaire et de calcul des coûts (48) et l’Outil de calcul des vies sauvées (49). Ces résultats seront utilisés pour soutenir les décisions et processus politiques et de planification.

Enfin, pour trianguler plus avant la validité de nos conclusions, nous comparerons nos estimations de référence et à 36 mois pour les domaines de la *portée*, de l’*efficacité* et de l’*adoption* aux EDS du Togo les plus récentes ou aux données de l’Enquête par grappe a indicateur multiple (MICS). Nous comparerons également les domaines de la *portée* et de l’*adoption* au RSSBC via les données de programmation recueillies au niveau de la communauté et de la formation sanitaire.

**Plan de diffusion**

Nous diffuserons de manière régulière les données de l’étude auprès des parties prenantes au Togo au niveau national, régional et communautaire, ainsi qu’auprès de la communauté internationale des professionnels de la santé publique, chercheurs et décideurs politiques. Le personnel de SI organise des forums semestriels avec les dirigeants locaux, le personnel des formations sanitaires du secteur public et les membres de la communauté afin de discuter de la mise en œuvre du RSSBC et de partager ses résultats. Sur la base de ces forums, SI et les partenaires du MS décideront ensemble d’adapter la stratégie de mise en œuvre et, si jugé nécessaire, l’intervention. Le personnel de SI participera également aux réunions mensuelles du MS au niveau du district afin de rester à jour dans les plans du MS et de partager ses résultats d’initiatives de RSSBC. Les résultats seront publiés dans des résumés de conférence et dans des revues évaluées par des pairs avec une préférence pour les publications disponibles publiquement en collaboration avec les partenaires du MS togolais.

**Discussion**

Nous avons décrit notre justification, la conception de l’étude et les détails de la stratégie de mise en œuvre en ce qui concerne cette conception hybride pragmatique de type II visant à servir de modèle pour les personnes intéressées par les études de mise en œuvre pragmatiques qui permettent une amélioration continue de l’intervention.

La conception de l’étude présente plusieurs restrictions, y compris des restrictions liées aux Etudes randomisées en grappes avec permutation séquentielle (ou Stepped-wedge cluster randomized trials). Il existe des inquiétudes liées à la confusion, au biais et aux tendances temporelles qui peuvent limiter la validité des résultats. Nous avons utilisé une ampleur d’effet modeste, une randomisation des groupes et un plan d’analyse pour atténuer ces limitations. Il existe des facteurs contextuels qui peuvent se révéler complexes à délimiter et qui peuvent influencer nos résultats primaires. Nous avons tenté de les minimiser au début de l’étude via la sélection des formations sanitaires.

Malgré ces limitations, cette étude permettra une évaluation rigoureuse, en situation réelle, à l’aide des mesures de l’efficacité et de la stratégie de mise en œuvre, tout en contribuant également à la création de connaissances visant à informer et à soutenir les stratégies sanitaires nationales. Une approche uniforme dans l’évaluation ne fonctionne pas pour la mise en œuvre d’interventions multiples et de leurs stratégies correspondantes, en particulier en situation réelle. Nous avons pour objectif de fournir une intervention efficace accompagnée d’une stratégie de mise en œuvre qui inclut des facteurs favorables et défavorables. Via cette requête scientifique dirigée et une évaluation combinant différentes méthodes, nous nous efforçons de contribuer à la création de connaissances et encourageons les partenariats afin d’améliorer la qualité et l’accès aux systèmes communautaires de santé au Togo et au-delà.

**Abréviation**

OC: Organisation Communautaire ; CHSL : Community Health Systems Lab (ou Laboratoire des systèmes communautaires de santé ; ASC : Agent de Santé Communautaire ; EDS: Enquête Démographique et de Sante ; PEV: Programme Elargi de vaccination ; RSSBC : Renforcement des systèmes de santé et à base communautaire ; PCIME : Prise en Charge Intégrée des Maladies de l’Enfant ; PCIME-C : Prise en Charge Intégrée des Maladies de l’Enfant au niveau Communautaire ; SI : Santé Intégrée ; LiST: Lives Saved Tool (ou Outil de calcul des vies sauvées); MICS: Enquête par grappes à indicateurs multiples ; MS : Ministère de la Santé et de l’Hygiène publique ; ONG : Organisation non gouvernementale ; RE-AIM: Reach, effectiveness, adoption, implementation, maintenance (ou : Portée, efficacité, adoption, mise en œuvre, maintien); SARA: Service Availability and Readiness Assessment (ou Évaluation de la préparation et de la disponibilité des services); ODD: Objectif de Développement Durable ; SW-CRT: Stepped-wedge cluster randomized trial (ou étude randomisée en grappe avec permutation séquentielle); OMS : Organisation mondiale de la santé

**Remerciements**

Nous exprimons notre reconnaissance aux partenaires et conseillers suivants pour leur soutien indéfectible dans le développement et la mise en œuvre de cette étude : les autorités sanitaires de la région de Kara et des préfectures de Bassar, Binah, Dankpen et Kéran ; le personnel de SI au Togo et à New York ; l’Albert Einstein College of Medicine et l’université de Washington.

**Contributions des auteurs**

Tous les auteurs ont contribué au développement de la conception de l’étude. MEL, KPF, HEJ, SCG, KDL, et LRH ont rédigé et revu le protocole de l’étude. MEL, KPF et HEJ ont développé le plan analytique et statistique. Tous les auteurs participeront à la mise en œuvre de l’étude et ont lu et approuvé le manuscrit final.

**Financement**

L’étude a été financée par SI. Aucun bailleur de fonds n’a joué un rôle dans la conception de cette étude.

**Disponibilité des données et du matériel**

Le protocole de l’étude associée et les outils de recueil des données seront disponibles sur demande auprès de l’auteur correspondant. Les jeux de données quantitatives sont disponibles auprès de l’auteur correspondant sur demande raisonnable après la réalisation des analyses primaires et la diffusion des résultats. Les ensembles de données qualitatives ne seront pas disponibles, puisqu’ils peuvent inclure des informations personnelles pouvant comporter l’identité de la participante.

**Approbation éthique et consentement à la participation**

Les approbations éthiques pour cette étude ont été obtenues auprès des comités d’évaluation institutionnels du ministère de la Santé togolais situé à Lomé, Togo (réf : CRBS/33/2017) et auprès de l’Albert Einstein College of Medicine de New York, aux États-Unis (réf : 039328). Le consentement éclairé sera obtenu auprès de toutes les participantes à l’étude âgées de plus de 18 ans et une autorisation parentale sera demandée aux participantes âgées de 15 à 17 ans. En cas de refus, ces personnes ne seront pas incluses dans l’étude.

**Consentement à la publication**

Sans objet.

**Intérêts concurrents**

Les auteurs déclarent ne pas avoir d’intérêts concurrents.

**Informations au sujet des auteurs**

^1^Community Health Systems Lab, Integrate Health/Santé Intégrée, Kara, Togo. ^2^ Service de pédiatrie, Albert Einstein College of Medicine, Bronx, NY, États-Unis. ^3^ Service de médecine familiale et sociale, Albert Einstein College of Medicine, Bronx, NY, États-Unis. ^5^Integrate Health/Santé Intégrée, Kara, Togo. ^6^ Service sanitaire régional de Kara, ministère de la Santé et de l’Hygiène publique, Kara, Togo. ^7^ Service de pédiatrie, faculté des sciences de la santé, université de Lomé, Lomé, Togo. ^9^Feinberg School of Medicine de la Northwestern University, Chicago, États-Unis

**Références :**

1. World Health Organization. World Health Statistics 2015. 2015.

2. Golding N, Burstein R, Longbottom J, Browne AJ, Fullman N, Osgood-Zimmerman A, et al. Mapping under-5 and neonatal mortality in Africa, 2000-15: a baseline analysis for the Sustainable Development Goals. Lancet (London, England) [Internet]. 2017 Nov 11 [cited 2018 Oct 22]; 390(10108):2171–82. Available from: http://www.ncbi.nlm.nih.gov/pubmed/28958464

3. Ministère de la Planification du Développement et de l’Aménagement du Territoire (MPDAT) M de la SM et II. Enquête Démographique et de Sante au Togo 2013 – 2014. Rockville, Maryland, USA ; 2015.

4. UN Inter-agency Group for Child Mortality Estimation (UN IGME). Country-specific under-five mortality rate, 2018 [Internet]. 2018 [cited 2018 Dec 1]. Available from: https://data.unicef.org/topic/child-survival/under-five-mortality/

5. Togo Ministry of Health. Togo Ministère de la Santé : Rapport Annuel de Performance Gestion 2014. 2014.

6. Togo Ministry of Health. Togo Ministère de la Santé : Principaux Indicateurs de Sante 2014. 2014.

7. Fiori K, Schechter J, Dey M, Braganza S, Rhatigan J, Houndenou S, et al. Closing the delivery gaps in pediatric HIV care in Togo, West Africa: using the care delivery value chain framework to direct quality improvement. AIDS Care - Psychol Socio-Medical Asp AIDS/HIV. 2016; 28(September 2017):29–33.

8. Fiori KP, Belli HM, Lauria ME, Hirschhorn LR, Schechter J, Hansman E, et al. Implementing an integrated community based health systems strengthening approach to improve HIV survival in Northern Togo. AIDS Care [Internet]. 2019 Jun 6 [cited 2019 Jul 8]; 1–9. Available from: https://www.tandfonline.com/doi/full/10.1080/09540121.2019.1626342

9. McCarthy KJ, Braganza S, Fiori K, Gbeleou C, Kpakpo V, Lopez A, et al. Identifying inequities in maternal and child health through risk stratification to inform health systems strengthening in Northern Togo. PLoS One. 2017; 12(3):1–19.

10. Arnold J, Samson M, Schechter J, Goodwin AS, Braganza S, Sesso GC, et al. Getting There: Overcoming Barriers to Reproductive and Maternal Health Services Access in Northern Togo-A Qualitative Study. World Med Heal Policy [Internet]. 2016 Sep 1 [cited 2019 Jul 30]; 8(3):223–44. Available from: http://doi.wiley.com/10.1002/wmh3.195

11. Johnson AD, Thomson DR, Atwood S, Alley I, Beckerman JL, Kon?? I, et al. Assessing early access to care and child survival during a health system strengthening intervention in Mali: A repeated cross sectional survey. PLoS One. 2013; 8(12).

12. Cometto G, Ford N, Pfaffman-Zambruni J, Akl EA, Lehmann U, McPake B, et al. Health policy and system support to optimise community health worker programmes: an abridged WHO guideline. Lancet Glob Heal [Internet]. 2018 Dec [cited 2019 Jul 30]; 6(12):e1397–404. Available from: http://www.ncbi.nlm.nih.gov/pubmed/30430994

13. O’Mara-Eves A, Brunton G, McDaid D, Oliver S, Kavanagh J, Jamal F, et al. Community engagement to reduce inequalities in health: a systematic review, meta-analysis and economic analysis [Internet]. Community engagement to reduce inequalities in health: a systematic review, meta-analysis and economic analysis. NIHR Journals Library; 2013 [cited 2019 Mar 18]. Available from: http://www.ncbi.nlm.nih.gov/pubmed/25642563

14. O’Mara-Eves A, Brunton G, Oliver S, Kavanagh J, Jamal F, Thomas J. The effectiveness of community engagement in public health interventions for disadvantaged groups: a meta-analysis. BMC Public Health [Internet]. 2015 Dec 12 [cited 2019 Mar 18]; 15(1):129. Available from: http://www.ncbi.nlm.nih.gov/pubmed/25885588

15. Kok MC, Dieleman M, Taegtmeyer M, Broerse JE, Kane SS, Ormel H, et al. Which intervention design factors influence performance of community health workers in low- and middle-income countries? A systematic review. Health Policy Plan [Internet]. 2015 Nov [cited 2019 Feb 9]; 30(9):1207–27. Available from: http://www.ncbi.nlm.nih.gov/pubmed/25500559

16. Yates R. Universal health care and the removal of user fees. Lancet (London, England) [Internet]. 2009 Jun 13 [cited 2019 Mar 15]; 373(9680):2078–81. Available from: http://www.ncbi.nlm.nih.gov/pubmed/19362359

17. Johri M, Ridde V, Heinmüller R, Haddad S. Estimation of maternal and child mortality one year after user-fee elimination: an impact evaluation and modelling study in Burkina Faso. Bull World Health Organ [Internet]. 2014 Oct 1 [cited 2018 Oct 31]; 92(10):706–15. Available from: http://www.who.int/entity/bulletin/volumes/92/10/13-130609.pdf

18. Calhoun LM, Speizer IS, Guilkey D, Bukusi E. The Effect of the Removal of User Fees for Delivery at Public Health Facilities on Institutional Delivery in Urban Kenya. Matern Child Health J [Internet]. 2017; 0(0):0. Available from: http://link.springer.com/10.1007/s10995-017-2408-7

19. Johnson AD, Thiero O, Whidden C, Poudiougou B, Diakité D, Traoré F, et al. Proactive community case management and child survival in periurban Mali. BMJ Glob Heal Mali BMJ Glob Heal. 2018; 33.

20. Schellenberg JRA, Adam T, Mshinda H, Masanja H, Kabadi G, Mukasa O, et al. Effectiveness and cost of facility-based Integrated Management of Childhood Illness (IMCI) in Tanzania. Lancet [Internet]. 2004 Oct 30 [cited 2019 Mar 18]; 364(9445):1583–94. Available from: https://www.sciencedirect.com/science/article/pii/S014067360417311X

21. Christopher JB, Le May A, Lewin S, Ross DA. Thirty years after Alma-Ata: a systematic review of the impact of community health workers delivering curative interventions against malaria, pneumonia and diarrhoea on child mortality and morbidity in sub-Saharan Africa [Internet]. 2011 [cited 2019 Mar 18]. Available from: http://www.human-resources-health.com/content/9/1/27

22. Lassi ZS, Bhutta ZA. Community-based intervention packages for reducing maternal and neonatal morbidity and mortality and improving neonatal outcomes. Cochrane Database Syst Rev [Internet]. 2015 Mar 23 [cited 2019 Mar 18];(3). Available from: http://doi.wiley.com/10.1002/14651858.CD007754.pub3

23. Amouzou A, Morris S, Moulton LH, Mukanga D. Assessing the impact of integrated community case management (iCCM) programs on child mortality: Review of early results and lessons learned in sub-Saharan Africa. J Glob Health [Internet]. 2014 Dec [cited 2019 Mar 18]; 4(2):020411. Available from: http://www.ncbi.nlm.nih.gov/pubmed/25520801

24. Gogia S, Sachdev HS. Home visits by community health workers to prevent neonatal deaths in developing countries: a systematic review. Bull World Health Organ [Internet]. 2010 Sep 1 [cited 2019 Mar 18]; 88(9):658–666B. Available from: http://www.ncbi.nlm.nih.gov/pubmed/20865070

25. Gogia S, Sachdev HPS. Home-based neonatal care by community health workers for preventing mortality in neonates in low- and middle-income countries: a systematic review. J Perinatol [Internet]. 2016 [cited 2019 Mar 18]; 36 Suppl 1(Suppl 1):S55-73. Available from: http://www.ncbi.nlm.nih.gov/pubmed/27109093

26. Dawson AJ, Buchan J, Duffield C, Homer CSE, Wijewardena K. Task shifting and sharing in maternal and reproductive health in low-income countries: a narrative synthesis of current evidence. Health Policy Plan [Internet]. 2014 May 1 [cited 2019 Mar 18]; 29(3):396–408. Available from: https://academic.oup.com/heapol/article-lookup/doi/10.1093/heapol/czt026

27. Tso LS, Best J, Beanland R, Doherty M, Lackey M, Ma Q, et al. Facilitators and barriers in HIV linkage to care interventions: a qualitative evidence review. AIDS [Internet]. 2016 [cited 2019 Mar 18]; 30(10):1639–53. Available from: http://www.ncbi.nlm.nih.gov/pubmed/27058350

28. Hirschhorn LR, Baynes C, Sherr K, Chintu N, Awoonor-Williams JK, Finnegan K, et al. Approaches to ensuring and improving quality in the context of health system strengthening: A cross-site analysis of the five African Health Initiative Partnership programs. BMC Health Serv Res. 2013; 13(SUPPL.2).

29. Manzi A, Magge H., Hedt-Gauthier B., Michaelis AP, Cyamatare FR, Nyirazinyoye L. Clinical mentorship to improve pediatric quality of care at the health centers in rural Rwanda: a qualitative study of perceptions and acceptability of health care workers. Hirschhorn LR, Ntaganira J. BMC Health Serv Res. 2014; 14.

30. Magge H, Anatole M, Cyamatare FR, Mezzacappa C, Nkikabahizi F, Niyonzima S, et al. Mentoring and quality improvement strengthen integrated management of childhood illness implementation in rural Rwanda. Arch Dis Child. 2015; 100(6):565–70.

31. Zulu JM, Kinsman J, Michelo C, Hurtig A-K. Integrating national community-based health worker programmes into health systems: a systematic review identifying lessons learned from low-and middle-income countries. BMC Public Health [Internet]. 2014 Dec 22 [cited 2019 Mar 18]; 14(1):987. Available from: https://bmcpublichealth.biomedcentral.com/articles/10.1186/1471-2458-14-987

32. Noordam AC, Carvajal-Velez L, Sharkey AB, Young M, Cals JWL. Care Seeking Behaviour for Children with Suspected Pneumonia in Countries in Sub-Saharan Africa with High Pneumonia Mortality. Patra J, editor. PLoS One. 2015 Feb; 10(2):e0117919.

33. Sunguya BF, Mlunde LB, Ayer R, Jimba M. Towards eliminating malaria in high endemic countries: the roles of community health workers and related cadres and their challenges in integrated community case management for malaria: a systematic review. Malar J [Internet]. 2017 [cited 2019 Mar 18]; 16:10. Available from: https://www.ncbi.nlm.nih.gov/pmc/articles/PMC5209914/pdf/12936_2016_Article_1667.pdf

34. World Health Organization. Service Availability and Readiness Assessment (SARA) An annual monitoring system for service delivery, version 2.1. Geneva; 2013.

35. Proctor EK, Powell BJ, Mcmillen JC. Implementation strategies: recommendations for specifying and reporting [Internet]. 2013 [cited 2019 Jun 13]. Available from: http://www.implementationscience.com/content/8/1/139

36. Curran GM, Bauer M, Mittman B, Pyne JM, Stetler C. Effectiveness-implementation hybrid designs: combining elements of clinical effectiveness and implementation research to enhance public health impact. Med Care [Internet]. 2012 Mar [cited 2018 Oct 30]; 50(3):217–26. Available from: http://www.ncbi.nlm.nih.gov/pubmed/22310560

37. Leeman J, Birken SA, Powell BJ, Rohweder C, Shea CM. Beyond “implementation strategies”: classifying the full range of strategies used in implementation science and practice. Implement Sci [Internet]. 2017 Dec 3 [cited 2019 Jun 16]; 12(1):125. Available from: http://implementationscience.biomedcentral.com/articles/10.1186/s13012-017-0657-x

38. Bernet AC, Willens DE, Bauer MS. Effectiveness-implementation hybrid designs: implications for quality improvement science. Bernet al Implement Sci [Internet]. 2012 [cited 2018 Oct 30];(8):2. Available from: http://www.implementationscience.com/content/8/S1/S2

39. Fetters MD, Curry LA, Creswell JW. Achieving Integration in Mixed Methods Designs—Principles and Practices. Health Serv Res [Internet]. 2013 [cited 2019 May 15]; 48(6 Pt 2):2134. Available from: https://www.ncbi.nlm.nih.gov/pmc/articles/PMC4097839/

40. Langlois E V, Montekio VB, Young T, Song K, Alcalde-Rabanal J, Tran N. Enhancing evidence informed policymaking in complex health systems: lessons from multi-site collaborative approaches. 2016 [cited 2019 Mar 11]; Available from: https://www.ncbi.nlm.nih.gov/pmc/articles/PMC4794922/pdf/12961_2016_Article_89.pdf

41. Wolfenden L, Williams CM, Wiggers J, Nathan N, Yoong SL. Improving the translation of health promotion interventions using effectiveness-implementation hybrid designs in program evaluations. Heal Promot J Aust [Internet]. 2016 Dec 1 [cited 2019 Jan 17]; 27(3):204–7. Available from: http://doi.wiley.com/10.1071/HE16056

42. Glasgow RE, Vogt TM, Boles SM. Evaluating the public health impact of health promotion interventions: the RE-AIM framework. Am J Public Health. 1999 Sep; 89(9):1322–7.

43. Glasgow RE, Estabrooks PE. Pragmatic Applications of RE-AIM for Health Care Initiatives in Community and Clinical Settings. Prev Chronic Dis [Internet]. 2018 Jan 4 [cited 2019 Apr 11]; 15:170271. Available from: http://www.cdc.gov/pcd/issues/2018/17_0271.htm

44. Glasgow RE, Klesges LM, Dzewaltowski DA, Estabrooks PA, Vogt TM. Evaluating the impact of health promotion programs: using the RE-AIM framework to form summary measures for decision making involving complex issues. Health Educ Res. 2006; 21(5):688–94.

45. Steckler A, McLeroy KR. The Importance of External Validity. Am J Public Health [Internet]. 2009 [cited 2019 Jan 31]; 98(1):9–10. Available from: https://www.ncbi.nlm.nih.gov/pmc/articles/PMC2156062/pdf/0980009.pdf

46. Mackenzie M, O’Donnell C, Halliday E, Sridharan S, Platt S. Do health improvement programmes fit with MRC guidance on evaluating complex interventions? BMJ [Internet]. 2010 Feb 1 [cited 2019 Jan 22]; 340:c185. Available from: http://www.ncbi.nlm.nih.gov/pubmed/20123834

47. Ghaffar A, Langlois E V, Rasanathan K, Peterson S, Adedokun L, Tran NT. Strengthening health systems through embedded research. Bull World Health Organ [Internet]. 2017 [cited 2019 Jan 22]; 95(2):87. Available from: http://www.ncbi.nlm.nih.gov/pubmed/28250505

48. Collins D, Jarrah Z. Community Health Planning and Costing Tool [Internet]. Management Sciences for Health; 2016 [cited 2018 Oct 22]. Available from: https://www.msh.org/resources/community-health-planning-and-costing-tool

49. Walker N, Friberg IK. Introduction: reporting on updates in the scientific basis for the Lives Saved Tool (LiST). BMC Public Health [Internet]. 2017 Nov 7 [cited 2018 Oct 22]; 17(S4):774. Available from: https://bmcpublichealth.biomedcentral.com/articles/10.1186/s12889-017-4735-4

50. Hemming K, Taljaard M, Grimshaw J. Introducing the new CONSORT extension for stepped-wedge cluster randomised trials. Trials [Internet]. 2019 Dec 18 [cited 2019 Jan 25]; 20(1):68. Available from: https://trialsjournal.biomedcentral.com/articles/10.1186/s13063-018-3116-3

51. Hemming K, Haines TP, Chilton PJ, Girling AJ, Lilford RJ. The stepped wedge cluster randomised trial: rationale, design, analysis, and reporting. BMJ. 2015 Feb; 350:h391.

52. Hayes RJ, Moulton LH. Cluster Randomised Trials. Boca Raton: Taylor & Francis Group, LLC; 2009.

53. Ministre de la Sante et de la Protection Sociale (MSPS). Plan National de Developpement Sanitaire 2017-2022. Lomé, Togo; 2017.

54. Kish L. A Procedure for Objective Respondent Selection within the Household. J Am Stat Assoc. 1949 Sep; 44(247):380–7.

55. Baio G, Copas A, Ambler G, Hargreaves J, Beard E, Omar RZ. Sample size calculation for a stepped wedge trial. Trials Baio al Trials. 2015; 16(16).

56. Belza B, Toobert D, Glasglow R. RE-AIM for Program Planning: Overview and Applications.

57. Graham W, Brass W, Snow RW. Estimating Maternal Mortality: The Sisterhood Method. Stud Fam Plann [Internet]. 1989 May [cited 2018 Nov 7]; 20(3):125. Available from: https://www.jstor.org/stable/1966567?origin=crossref

58. World Health Organization, Division of Reproductive Health. The Sisterhood Method for Estimating Maternal Mortality: Guidance notes for potential users. Geneva; 1997.

59. Damschroder LJ. Fostering implementation of health services research findings into practice: a consolidated framework for advancing implementation science. Implement Sci [Internet]. 2009; 1–15. Available from: http://www.implementationscience.com/content/4/1/50
